# The RANK/RANKL axis controls vascular dynamics in the bone marrow

**DOI:** 10.1073/pnas.2425366122

**Published:** 2025-11-03

**Authors:** Takeshi Kaneko, Shinya Yari, Junichi Kikuta, Yoshiki Omatsu, Shigeto Seno, Sumire Kikuchi, Kazuma Sato, Kentaro Fujii, Takao Sudo, Tetsuo Hasegawa, Kunimaro Furuta, Qianqian Guo, Samar H. Ibrahim, Kosuke Muraoka, Yoshiaki Okada, Yoshiaki Kubota, Daisuke Okuzaki, Yasuhiro Kobayashi, Atsushi Kumanogoh, Nobuyuki Udagawa, Takashi Nagasawa, Josef M. Penninger, Masaru Ishii

**Affiliations:** ^a^Department of Immunology and Cell Biology, Graduate School of Medicine and Frontier Biosciences, The University of Osaka, Osaka 565-0871, Japan; ^b^Department of Respiratory Medicine and Clinical Immunology, Graduate School of Medicine, The University of Osaka, Osaka 565-0871, Japan; ^c^World Premier International Research Center Initiative Immunology Frontier Research Center, The University of Osaka, Osaka 565-0871, Japan; ^d^Laboratory of Bioimaging and Drug Discovery, National Institutes of Biomedical Innovation, Health and Nutrition, Osaka 567-0085, Japan; ^e^Laboratory of Stem Cell Biology and Developmental Immunology, Graduate School of Medicine and Frontier Biosciences, The University of Osaka, Osaka 565-0871, Japan; ^f^Department of Bioinformatic Engineering, Graduate School of Information Science and Technology, The University of Osaka, Osaka 565-0871, Japan; ^g^Division of Gastroenterology and Hepatology, Mayo Clinic, Rochester, MN 55905; ^h^Graduate School of Pharmaceutical Sciences, The University of Osaka, Osaka 565-0871, Japan; ^i^Department of Anatomy, Keio University School of Medicine, Tokyo 160-8582, Japan; ^j^Genome Information Research Center, Research Institute for Microbial Diseases, The University of Osaka, Osaka 565-0871, Japan; ^k^Department of Biochemistry, Matsumoto Dental University, Nagano 399-0704, Japan; ^l^Helmholtz Centre for Infection Research, Braunschweig, Lower Saxony 38124, Germany; ^m^Department of Laboratory Medicine, Medical University of Vienna, Vienna 1090, Austria; ^n^Department of Medical Genetics, Life Sciences Institute, University of British Columbia, Vancouver, BC V6T 1Z3, Canada; ^o^Life-omics Research Division, Institute for Open and Transdisciplinary Research Initiative, The University of Osaka, Osaka 565-0871, Japan

**Keywords:** RANK/RANKL axis, vascular permeability, bone marrow, intravital imaging, bone metabolism

## Abstract

Receptor activator of nuclear factor kappa B ligand (RANKL) is an essential cytokine that induces osteoclastic differentiation by monocyte-macrophage lineage precursors. Here, we showed that in addition to its conventional action, RANKL controls vascular permeability in the bone marrow, where it facilitates the mobilization of hematopoietic monocytic cells, including osteoclast precursors, and resultantly regulates bone metabolism. RANK, a cognate receptor for RANKL, is abundantly expressed in sinusoidal endothelial cells and controls vascular permeability by regulating the expression patterns of intercellular adhesion molecule 1 and vascular cell adhesion molecule 1. High RANKL expression was detected in perivascular C–X–C motif chemokine ligand 12-abundant reticular (CAR) stromal cells. Specific deletion of RANKL expression in CAR cells abrogated the vascular leakage, suggesting that perivascular RANKL is responsible for controlling permeability. In summary, our study revealed a role for RANK/RANKL signaling as a gatekeeper of bone marrow sinusoids in vivo.

## Significance

Receptor activator of nuclear factor kappa B ligand (RANKL) is a multifunctional cytokine and has been attracting high levels of interest in broader fields of biomedical sciences. In this study, we showed that RANKL from C–X–C motif chemokine ligand 12 (CXCL12)-abundant reticular (CAR) stromal cells control vascular permeability and transmigration of monocytoid cells in the bone marrow. We found that RANK expressed on bone marrow endothelial cells regulates vascular permeability and cell migration by upregulating adhesion molecules, thereby contributing to the control of bone metabolism. Overall, this study demonstrates the enhanced migration of osteoclast precursors into the bone marrow under the direction of the RANK/RANKL axis. These events represent a critical point in the regulation of bone homeostasis.

The vascular network supplies essential nutrients and oxygen, removes waste products, and relocates immune cells. The bone marrow is a highly vascularized tissue, and blood vessels passing through the bone marrow are fenestrated. Hematopoiesis occurs in bone marrow, where many cellular components of blood are produced and organized by the immune system, and various types of bone marrow cells continuously enter and egress from bone marrow parenchyma (1). Additionally, monocyte/macrophage lineage cells including osteoclast precursors migrate across the capillary endothelium and are controlled by several chemokines and mediators, such as sphingosine-1-phosphate and C–X–C motif chemokine ligand 12 (CXCL12) (2, 3). The bone marrow vasculature must be sufficiently permeable to relocate immune cells and allow passage of several molecules and plasma proteins. However, the actual mechanisms controlling vascular permeability in bone marrow spaces have remained unclear, partially because of the lack of practical methodologies for evaluating the leakage in vivo.

Intravital multiphoton imaging is a powerful tool for dissection of cellular/molecular dynamics in intact tissues and organs in living animals (4–7). The methods for intravital visualization of live bone tissues developed by our group have enabled us to analyze the behaviors and interactions of bone-destroying osteoclasts (8, 9) and their precursors (2, 3), as well as bone-replenishing osteoblasts (10–12). This intravital imaging methodology, in which the systemic circulation remains intact, can potentially be used to analyze vascular integrity and transluminal cell migration precisely.

In this study, using a higher-resolution multiphoton microscopy system, we successfully visualized vascular leakage and the spontaneous transmigration of monocyte/macrophage lineage cells in the sinusoidal capillaries of bone marrow spaces in vivo. Our results showed that transmigration rates are significantly higher in the bone marrow than in other blood capillaries, such as the skin. Furthermore, we were able to demonstrate a function for receptor activator of nuclear factor κB ligand (RANKL), along with its previously described functions in osteoclast differentiation. Specifically, we found that RANKL plays a critical role in controlling the high vascular permeability of bone marrow sinusoids and in extravasation of hematopoietic cells, including osteoclast precursors; thus, it serves as a unique point of control in bone homeostasis.

## Results

### Intravital Imaging of High Vascular Permeability in the Bone Marrow.

**Fig. 1.**
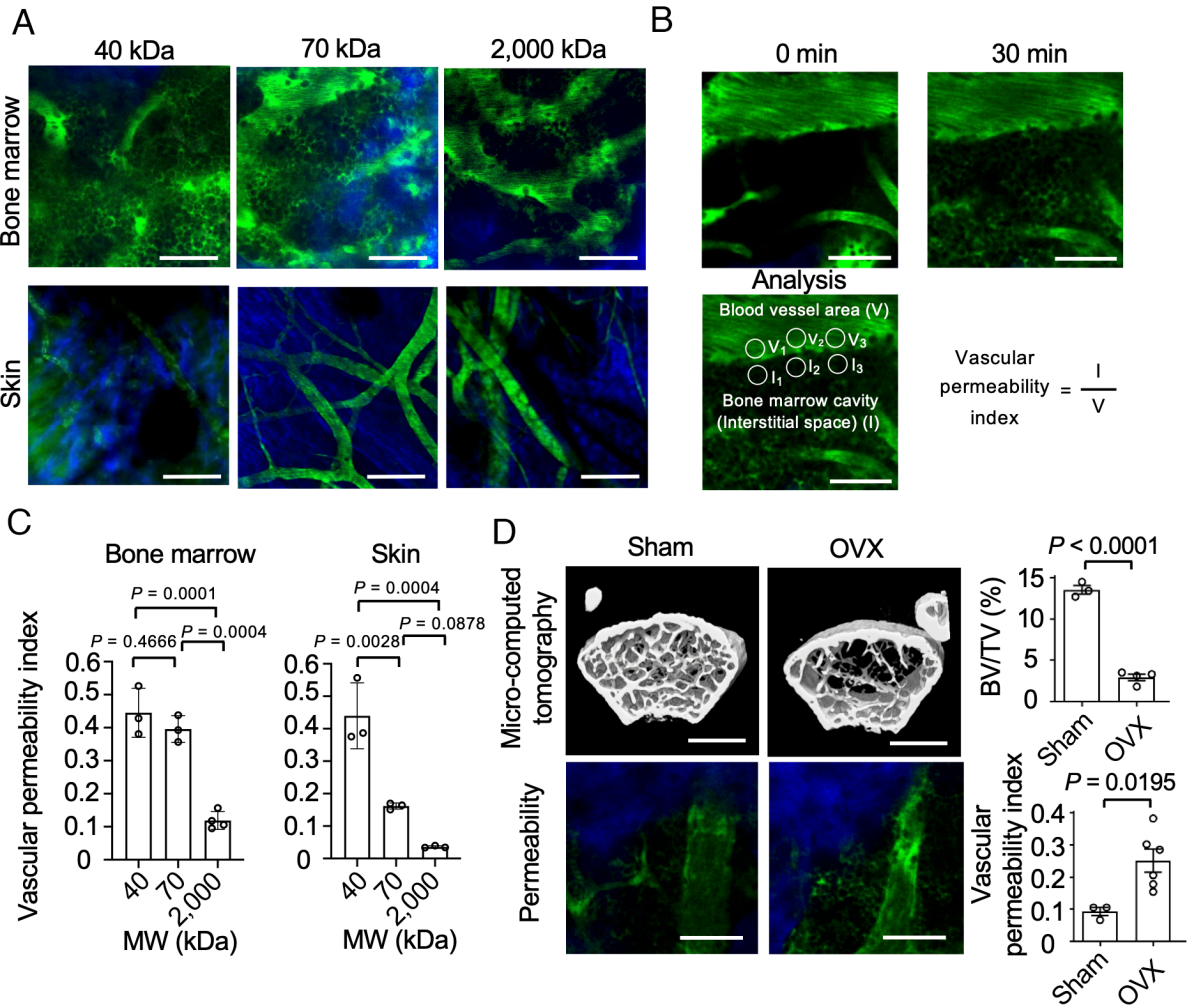
Intravital imaging of high vascular permeability in the bone marrow. (*A*) Representative intravital multiphoton images of mouse bone tissues (*Upper*) and skin (*Lower*) from wild-type (WT) mice injected with FITC-conjugated dextran of different molecular masses (40, 70, and 2,000 kDa) (n = 3 biological replicates per group). Blood vessels are shown in green; bones in the bone marrow (*Upper*) or collagen fibers in the skin (*Lower*) are shown in blue. [Scale bar: 50 μm (*Upper*) and 100 μm (*Lower*).] (*B*) Representative intravital multiphoton images of bone tissues from WT mice before and after intravenous injection of 70 kDa FITC-conjugated dextran (*Upper*), and corresponding analysis of vascular permeability in the bone marrow (*Lower*). The vascular permeability index was defined as the ratio of the mean fluorescence intensity in the bone marrow cavity (interstitial space) to the mean fluorescence intensity in the blood vessel area. (*C*) Summary of the vascular permeability index in the bone marrow and the skin (n = 3 biological replicates per group). (*D*) Representative micro-computed tomography images of the femurs of sham-operated mice (*Left*) and ovariectomized (OVX) mice (*Right*) (sham group: n = 3; ovariectomized group: n = 4 biological replicates). (Scale bar: 1 mm.) Summary of the trabecular bone volume to total bone volume (BV/TV) ratio (sham group: n = 3; OVX group: n = 4 biological replicates). Representative intravital multiphoton images of bone tissues in sham-operated mice (*Left*) and OVX mice (*Right*) (sham group: n = 3; OVX group: n = 6 biological replicates). Blood vessels are shown in green, and bones are shown in blue. (Scale bar: 50 μm.) Summary of the vascular permeability index (sham group: n = 3; OVX group: n = 6 biological replicates). Data are presented as the mean ± SEM. Statistical significance was determined by one-way ANOVA with Tukey’s test in (*C*) and a two-tailed unpaired *t* test in (*D*).

First, we estimated vascular permeability by intravenously injecting fluorescein isothiocyanate (FITC)-conjugated dextran of different molecular masses (40, 70, and 2,000 kDa) and then monitoring vessel leakage with a multiphoton microscope. The results demonstrated that 40 and 70 kDa dextrans were easily permeable, whereas 2,000 kDa dextrans showed reduced permeability (Fig. 1*A* and Movie S1). For comparison, vascular leakage was evaluated in the blood vessels of the skin (13). The leakage of dextran proteins >40 kDa from bone marrow but not skin vessels (Fig. 1*A*) demonstrated higher vascular permeability within the bone marrow. Vascular permeability was quantified by calculating the vascular permeability index, defined as the ratio of the mean fluorescence intensity in the bone marrow cavity (interstitial space) to that of the blood vessel area; a higher ratio indicated greater leakage of dextran from the vessels (Fig. 1*B*). The permeability index results indicated that capillaries were more permeable in the bone marrow than in the skin (Fig. 1*C*). Bone marrow vascular permeability under pathological conditions was examined in ovariectomized mice, which served as an animal model of postmenopausal osteoporosis. Osteoporosis was confirmed by measuring bone mineral density (BMD) (Fig. 1*D*). The results showed that ovariectomy (OVX) significantly increased vascular permeability in the bone marrow and decreased BMD (Fig. 1*D*).

### RANKL-Dependent Control of Vascular Permeability and the Recruitment of Monocyte/Macrophage Lineage Cells in Bone Marrow Sinusoids.

**Fig. 2.**
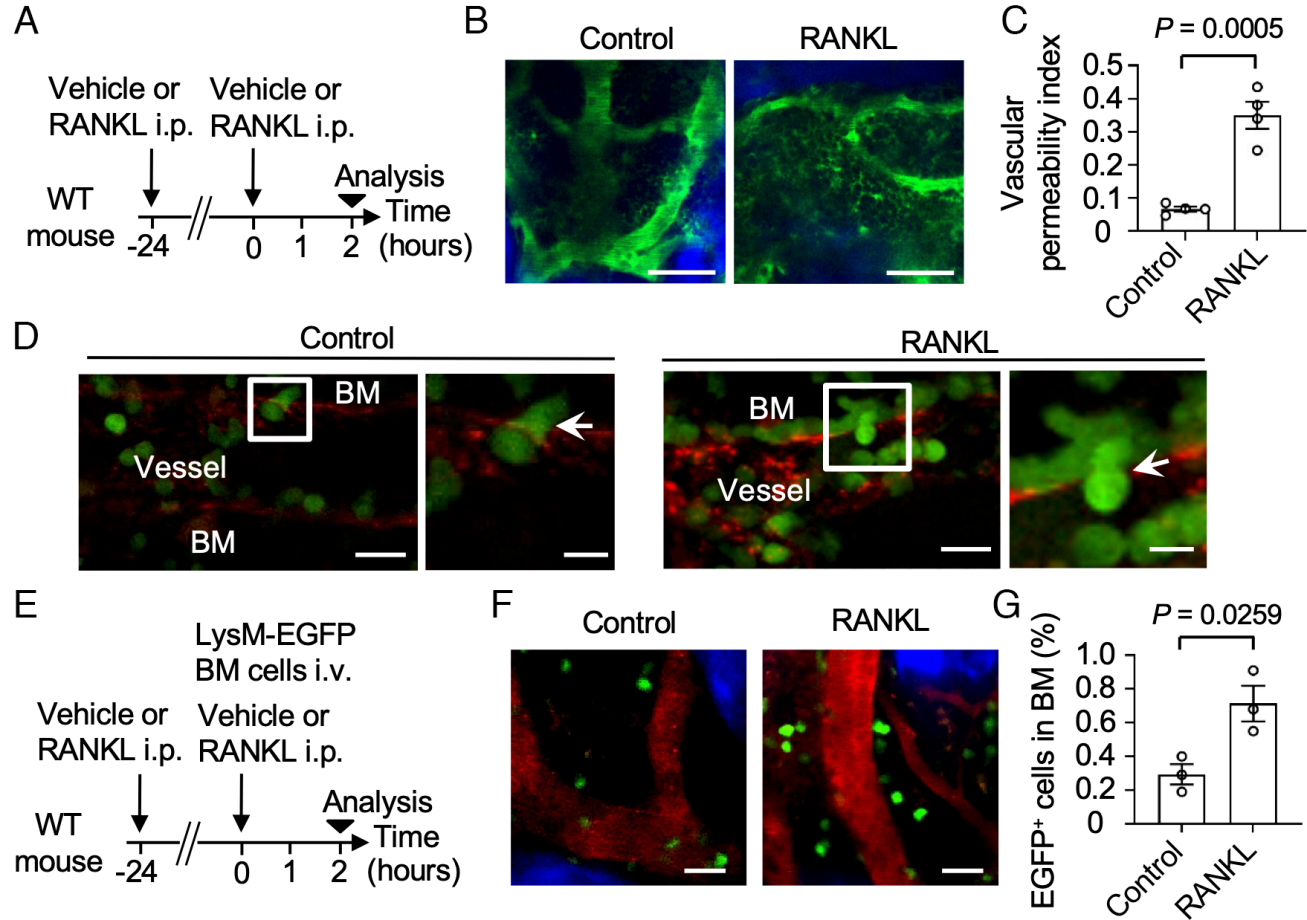
RANKL-dependent control of vascular permeability and the recruitment of monocyte/macrophage lineage cells in the bone marrow in vivo. (*A*) Experimental design. WT mice were used. Soluble RANKL (1 mg/kg) or vehicle was intraperitoneally injected at 24-h intervals for 2 d, and mice were analyzed 24 h later. (*B*) Representative images of intravital multiphoton imaging of the bone tissues of untreated (*Left*) and RANKL-treated (*Right*) WT mice after the injection of 2,000 kDa FITC-conjugated dextran (n = 4 biological replicates per group). Blood vessels are shown in green, and bones are shown in blue. (Scale bar: 50 μm.) (*C*) Vascular permeability index determined from (*B*) (n = 4 biological replicates per group). (*D*) Representative images of intravital multiphoton imaging of bone tissues from LysM-EGFP mice treated (*Right*) or not treated (*Left*) with RANKL after injection with isolectin Alexa Fluor 594 (n = 5 biological replicates per group). The experiment was performed five times with similar results. Blood vessel walls are shown in red, and EGFP-positive cells are shown in green. [Scale bars: 20 μm (*Left*), 10 μm (*Right*).] (*E*) Experimental design. WT mice were intraperitoneally injected with soluble RANKL (2 mg/kg) or vehicle at 24-h intervals for two consecutive days. WT mice were injected with bone marrow cells isolated from LysM-EGFP mice, and the transmigration frequency of EGFP-positive cells into sinusoidal capillaries of the bone marrow space was evaluated after 2 h. (*F*) Representative images of intravital multiphoton imaging of bone tissues of untreated (*Left*) and RANKL-treated (*Right*) WT mice injected with 70 kDa Texas Red-conjugated dextran (n = 4 biological replicates per group). Blood vessels are shown in red, bones are shown in blue, and EGFP-positive cells are shown in green. (Scale bar: 50 μm.) (*G*) Flow cytometry shows a significant increase in EGFP-positive cells in the bone marrow of mice with RANKL-induced osteoporosis (n = 3 biological replicates per group). Data are presented as the mean ± SEM. Statistical significance was determined by a two-tailed unpaired *t* test in (*C* and *G*).

A previous study showed that estrogen depletion by OVX or menopause enhances RANKL expression in osteoblasts and osteocytes, stimulating aberrant osteoclast differentiation and leading to osteoporosis (14). In the present study, RANKL-dependent control of bone marrow vascular permeability in vivo was investigated by examining the effects of systemic administration of recombinant RANKL (15). The leakage of 2,000 kDa dextran 48 h after RANKL administration suggested that increased vascular permeability was associated with osteoporosis (Fig. 2 *A*–*C*). RANKL-induced vascular permeabilization was also demonstrated in vitro through a decrease in the transendothelial electrical resistance (TEER) of RANKL-treated cultured microvascular endothelial cells (*SI Appendix*, Fig. S1 *A* and *B*). Additionally, the mice treated with RANKL resulted in the leakage of 70 kDa dextran from vessels in the skin, indicating that RANKL may also influence vascular permeability in the skin. However, the leakage of 2,000 kDa dextran after RANKL administration was not observed in the skin (*SI Appendix*, Fig. S2 *A* and *B*). Direct evidence of the relocation of immune cells across the vessel walls of the bone marrow cavity was acquired in observations of the transluminal migration of cells positive for enhanced green fluorescent protein (EGFP) under the control of the lysozyme M promoter (LysM-EGFP), representing neutrophils and monocyte/macrophage lineage cells (16). The number of transluminally migrating LysM-EGFP-positive cells was higher under RANKL administration than in the control condition (Fig. 2*D* and Movie S2). To assess the rate of transmigration of EGFP-positive cells into the sinusoidal capillaries of the bone marrow spaces, wild type (WT) mice were injected with LysM-EGFP mouse bone marrow cells and treated with RANKL (Fig. 2*E*). Intravital imaging and flow cytometric analysis showed a significant increase in EGFP-positive cells in the bone marrow under RANKL stimulation (Fig. 2 *F* and *G*), suggesting increased vascular permeability and the preferential recruitment of monocyte/macrophage lineage cells (e.g., osteoclast precursors) (17) into the bone marrow spaces as a feature of osteoporosis. A similar pattern was observed in an imaging study in which LysM-EGFP-positive cells were transferred into OVX-treated mice (*SI Appendix*, Fig. S3 *A* and *B*). These results are consistent with the findings of a previous study, in which the recruitment of circulating osteoclast precursor monocytes into the bone marrow cavities was enhanced by bone-resorptive states, resulting in an increased number of differentiated mature osteoclasts in bone (18). The versatile permeability of the bone marrow facilitates the delivery of osteoclast precursor monocytes, such that they are able to contribute locally to bone resorption.

### RANK Expressed on Bone Marrow Endothelial Cells (BMECs) Controls Cell Migration by Increasing Adhesion Molecules.

**Fig. 3.**
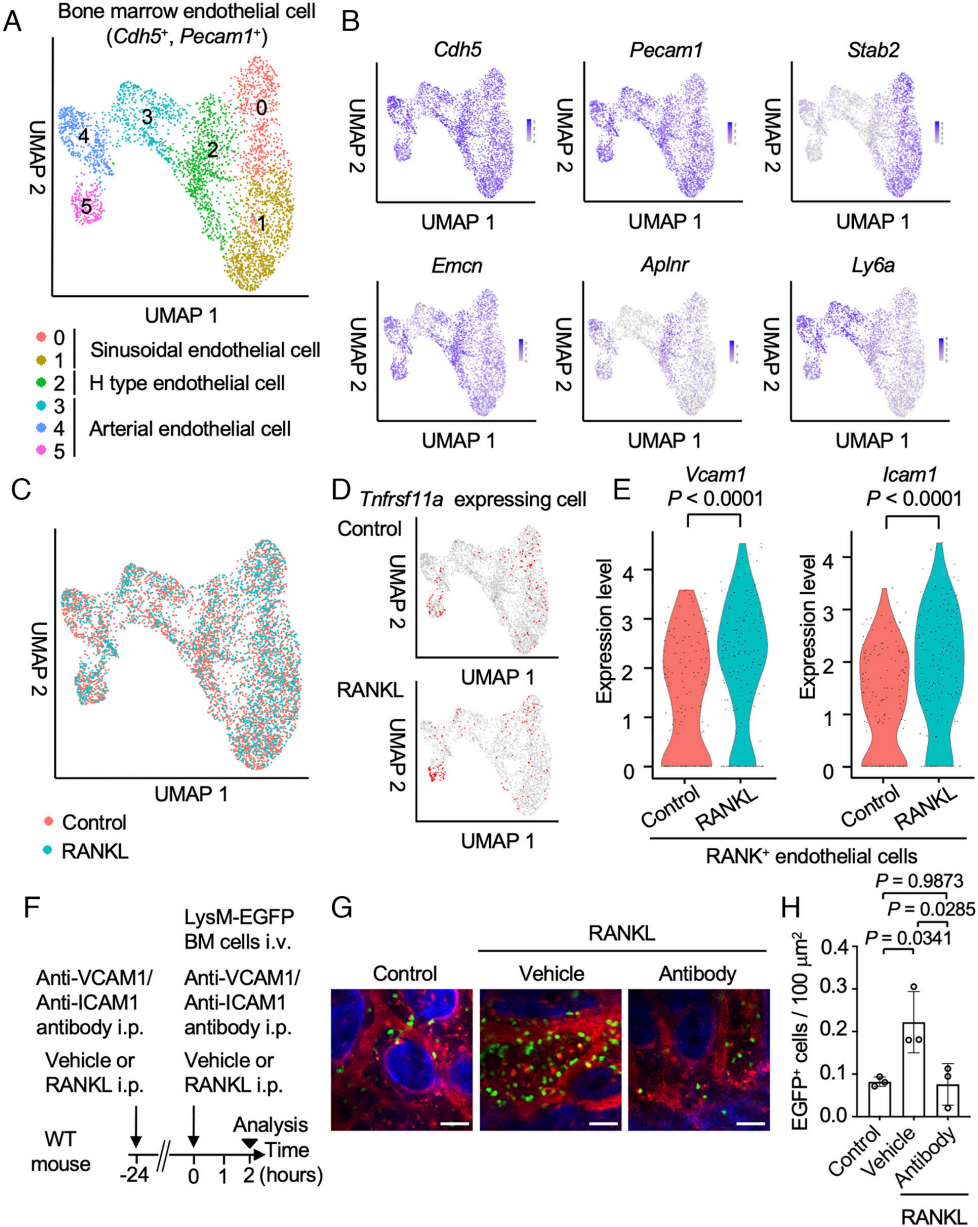
RANK expressed on BMECs controls cell migration by increasing adhesion molecules. (*A*) Uniform Manifold Approximation and Projection (UMAP) showing the clustering result of the BMECs (*Cdh5*^+^, and *Pecam1*^+^) integrated RANKL treatment and control group. (*B*) Feature plots of the gene markers of general endothelial cell (*Cdh5*, *Pecam1*), sinusoidal (*Stab2*), H-type (*Emcn*, *Aplnr*), and arterial (*Ly6a*) endothelial cell. (*C*) Feature plot showing all cells derived from control or RANKL group. (*D*) Feature plots presenting the RANK positive BMECs in each group. The RANK-positive BMECs (expression level: *Tnfrsf11a* > 0) were shown in red. (*E*) Violin plots showing of gene expression of *Vcam1* (*Left*) and *Icam1* (*Right*) in RANK positive BMECs of control and RANKL treatment group. (*F*) Experimental design. WT mice were intraperitoneally injected with soluble RANKL (2 mg/kg) or vehicle at 24-h intervals for two consecutive days. WT mice also received intraperitoneal injections of 0.2 mg/mouse of anti-VCAM-1 and anti-ICAM-1 neutralizing antibodies or vehicle control at 24-h intervals for two consecutive days. WT mice were injected with bone marrow cells isolated from LysM-EGFP mice, and the transmigration frequency of EGFP-positive cells into sinusoidal capillaries of the bone marrow space was evaluated after 2 h. (*G*) Representative intravital multiphoton images of bone tissues from untreated mice (*Left*), mice treated with RANKL (*Middle*), and mice treated with RANKL plus anti-VCAM-1 and anti-ICAM-1 antibodies (*Right*). WT mice were intravenously injected with 70 kDa Texas Red-conjugated dextran (n = 3 biological replicates per group). Blood vessels are shown in red, bones are shown in blue, and EGFP-positive cells are shown in green. (Scale bar: 50 μm.) (*H*) Summary of the number of EGFP-positive cells in the bone marrow cavity. Data are presented as the mean ± SEM. Statistical significance was determined by a Wilcoxon rank sum test in (*E*), and one-way ANOVA with Tukey’s test in (*H*).

To investigate how RANKL impacts BMECs, we isolated CD31^+^ CD45^−^ TER119^−^ bone marrow cells from RANKL-treated mice and control mice using flow cytometry. These cells underwent single-cell RNA sequencing (scRNA-seq), with *Cdh5*^high^ *Pecam1*^high^ cells identified as BMECs (Fig. 3*A* and *SI Appendix*, Fig. S4 *A* and *B*) (19–21). Among these, *Stab2*^high^ cells were categorized as sinusoidal endothelial cells, *Ly6a*^high^ as arterial endothelial cells, and *Emcn*^high^ *Aplnr*^high^ as H-type vessels (Fig. 3*B*). We identified BMECs expressing the receptor activator of nuclear factor κB (RANK, encoded by *Tnfrsf11a*) (Fig. 3 *C* and *D*). We further explored functional differences between RANK-negative and RANK-positive BMECs by Gene Ontology analysis (*SI Appendix*, Fig. S4*C*). RANK-positive BMECs showed downregulation in cell junction assembly and upregulation in cell adhesion and leukocyte migration compared to RANK-negative BMECs. Additionally, we compared the effects of RANKL stimulation with the control group (*SI Appendix*, Fig. S5 *A* and *B*). The analysis revealed an increase in genes associated with leukocyte migration and nitric oxide processes in RANK-positive BMECs following RANKL treatment (*SI Appendix*, Fig. S5*A*). Furthermore, the expression of intercellular adhesion molecule 1 (*ICAM1*) (22) and vascular cell adhesion molecule 1 (*VCAM1*), which facilitate the extravasation of leukocytes (23), was upregulated in RANK-positive BMECs due to RANKL stimulation (Fig. 3*E* and *SI Appendix*, Figs. S5*B*, S6, and S7). To determine whether ICAM1 and VCAM1 contribute to the RANKL-induced increase in vascular permeability, we administered neutralizing antibodies against these molecules. The enhanced migration of osteoclast precursor lineage cells from blood vessels into the bone marrow cavity, observed following RANKL treatment, was markedly reduced in mice receiving the neutralizing antibodies (Fig. 3 *F*–*H*). To further validate these findings, we generated endothelial cell-specific VCAM1 conditional knockout (KO) mice by crossing VCAM1-floxed mice with VE-cadherin (*Cdh5*)-CreERT2 mice (VCAM1_Cdh5_^−/−^: *Cdh5*-CreERT2×VCAM1^f/f^). Similarly, the RANKL-driven recruitment of osteoclast precursors into the bone marrow cavity was significantly reduced in VCAM1_Cdh5_^−/−^ mice (*SI Appendix*, Fig. S8). Together, these results indicate that RANKL enhances the migration of osteoclast precursor cells from the vasculature to the bone marrow cavity through a mechanism dependent on ICAM1 and VCAM1.

### RANK Expressed on BMECs Controls Vascular Permeability and Bone Metabolism.

**Fig. 4.**
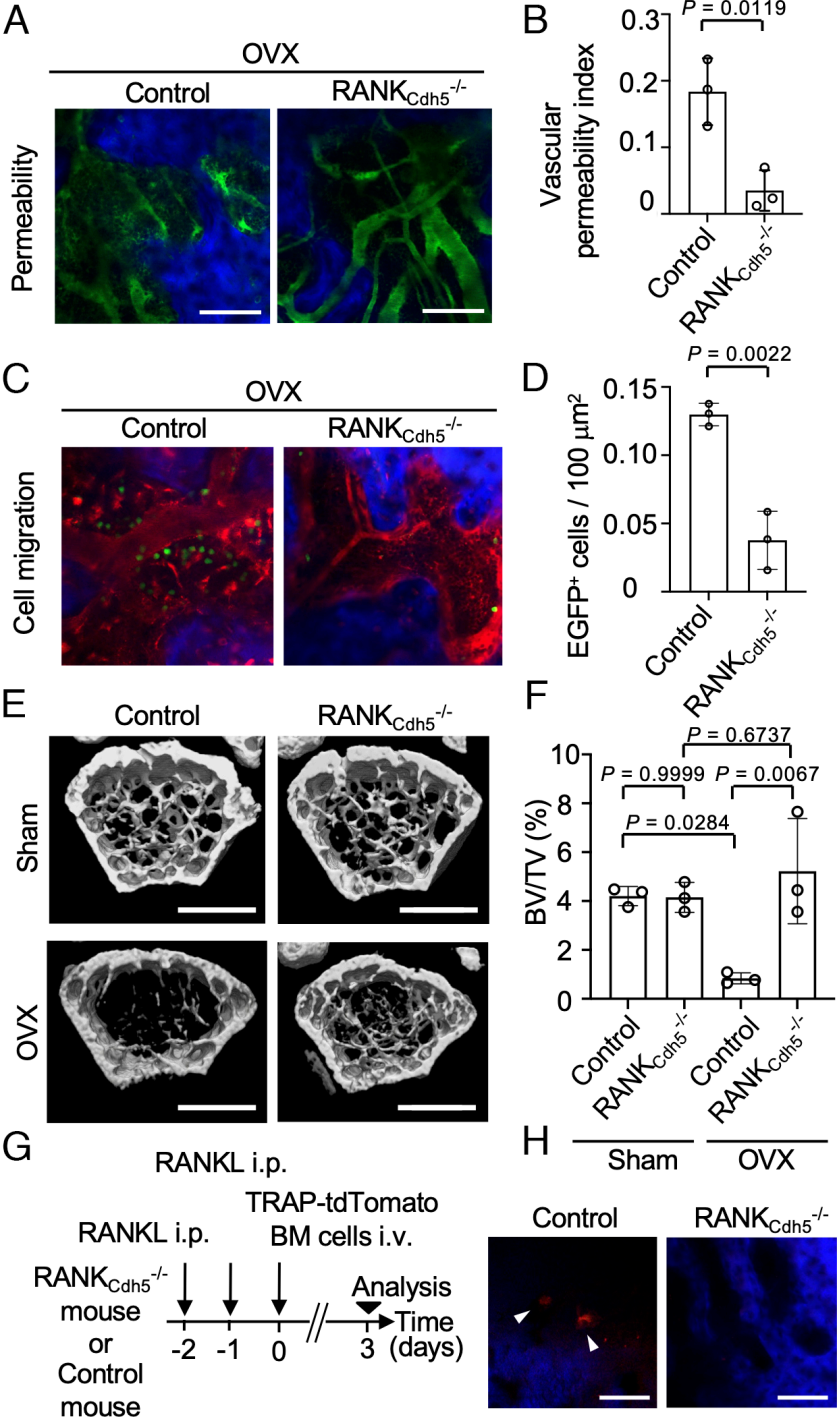
RANK expressed on BMECs controls vascular permeability and bone metabolism. (*A*) Representative intravital multiphoton images of bone marrow in endothelial cell-specific RANK conditional knockout mice (RANK_Cdh5_^−/−^: *Cdh5*-CreERT2 × RANK^f/f^) and control mice (RANK^f/f^) injected with FITC-conjugated dextran of molecular masses 2,000 kDa after OVX. Blood vessels are shown in green; bones in the bone marrow are shown in blue. (Scale bar: 50 μm.) (*B*) Summary of the vascular permeability index in bone marrow (n = 3 biological replicates per group). (*C*) Representative images of intravital multiphoton imaging of bone tissues in RANK_Cdh5_^−/−^ or control mice 1 mo after OVX, which were injected with bone marrow cells isolated from LysM-EGFP mice (n = 3 biological replicates per group). Mice were intravenously injected with 70 kDa Texas Red-conjugated dextran. Blood vessels are shown in red, bones are shown in blue, and EGFP-positive cells are shown in green. (Scale bar: 50 μm.) (*D*) Summary of the number of EGFP-positive cells in the bone marrow cavity area. (*E*) Representative micro-computed tomography images of femurs of sham-operated mice (*Upper*) and OVX mice (*Lower*) in RANK_Cdh5_^−/−^ or control mice. (Scale bar: 1 mm.) (*F*) Summary of the BV/TV ratio determined from images in (*E*) (n = 3 biological replicates per group). (*G*) Experimental design. RANK_Cdh5_^−/−^ or control mice were injected with the bone marrow cells including osteoclast precursors derived from TRAP-tdTomato mice, followed by imaging the bone marrow 3 d later. (*H*) Representative intravital multiphoton images showing the osteoclasts differentiated from the circulating osteoclast precursors. In control mice, TRAP-tdTomato positive osteoclasts differentiated from the circulating osteoclast precursors existed in the bone marrow (arrowhead), whereas RANK_Cdh5_^−/−^ mice did not have it. The experiment was performed three times with similar results. TRAP-tdTomato is shown in red. Bones in the bone marrow are shown in blue. (Scale bar indicates 100 µm.) Data are presented as the mean ± SEM. Statistical significance was determined by a two-tailed unpaired *t* test in (*B* and *D*), and one-way ANOVA with Tukey’s test in (*F*).

The impact of RANK expressed in BMECs was investigated using KO mice with RANK deletion in endothelial cells, achieved by crossing RANK-floxed mice with *Cdh5*-CreERT2 mice (RANK_Cdh5_^−/−^: *Cdh5*-CreERT2×RANK^f/f^) (24). We assessed whether OVX increased vascular permeability in RANK_Cdh5_^−/−^ mice compared to control mice. Results indicated that vascular permeability was not increased in RANK_Cdh5_^−/−^ mice after OVX (Fig. 4 *A* and *B*). Additionally, the enhanced migration of osteoclast precursor lineage cells from blood vessels into the bone marrow cavity, observed following OVX, was reduced in RANK_Cdh5_^−/−^ mice (Fig. 4 *C* and *D*). To clarify the importance of vascular-derived osteoclast precursor cells in bone resorption, we ovariectomized endothelial cell-specific RANK-deficient mice and then measured the bone morphology of their femurs. As shown in the figure, OVX led to a significant decrease in bone volume (BV/TV) in control mice. However, in endothelial RANK-deficient mice, bone volume was maintained at levels comparable to sham-operated mice, indicating protection against bone loss (Fig. 4 *E* and *F*). In addition, the osteoclast surface area (Oc.s/BS) and osteoclast number (N.Oc/BS) were increased in OVX control mice, consistent with enhanced bone resorption (*SI Appendix*, Fig. S9). In contrast, these changes were abolished in the endothelial RANK-deficient mice (*SI Appendix*, Fig. S9). Taken together, these results indicate that deletion of RANK in endothelial cells prevents OVX-induced bone loss. This highlights the crucial role of vascular-derived osteoclast precursor cells in the process of bone resorption. To determine the effect of RANK deficiency in BMECs on osteoclast differentiation from circulating osteoclast precursors, RANK_Cdh5_^−/−^ mice treated with RANKL were injected with bone marrow cells from the mice expressing the tdTomato reporter in the cytosol of mature osteoclasts (TRAP-tdTomato transgenic mice) (8, 9). Three days later, we found the tdTomato-labeled mature osteoclasts in the bone marrow of control mice, whereas we did not observe tdTomato-labeled mature osteoclasts in RANK_Cdh5_^−/−^ mice (Fig. 4 *G* and *H*). This suggests that RANK-positive BMECs regulate the entry of circulating osteoclast precursors into the bone marrow and maintain a critical balance in bone metabolism.

### The Analysis of RANKL Expression in the Bone Marrow.

**Fig. 5.**
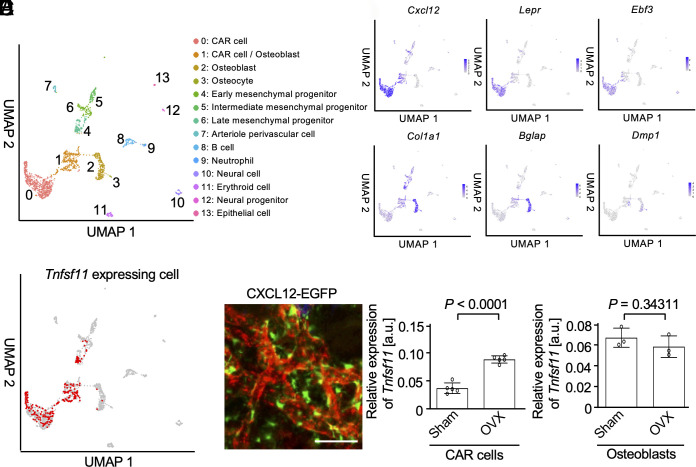
The analysis of RANKL expression in the bone marrow. (*A*) Clustering analysis showing the scRNA-seq data of mesenchymal cells in the bone marrow. The details of identifying the cell type were shown in *SI Appendix*, Fig. S11*A*. (*B*) Feature plots of the representative gene markers of CAR cell (*Cxcl12*, *Lepr*, *Ebf3*), osteoblast (*Col1a1*, *Bglap*), and osteocyte (*Dmp1*). (*C*) Feature plot presenting the RANKL positive cells. The RANKL positive cells (expression level: *Tnfsf11* > 0) were shown in red. (*D*) Representative intravital multiphoton images of calvarial bones in CXCL12-EGFP mice. CAR cells are shown in green. Blood vessels are shown in red. Scale bar: 50 μm. (*E*) RANKL messenger RNA (mRNA) expression levels in CAR cells (n = 5 biological replicates per group). (*F*) RANKL mRNA expression levels in osteoblasts (n = 3 biological replicates per group). Data are presented as the mean ± SEM. Statistical significance was determined by a two-tailed unpaired *t* test in (*E* and *F*).

Next, we aimed to identify the source of endogenous RANKL controlling sinusoidal vascular permeability. Osteoblasts represent a major source of RANKL supporting osteoclastogenesis (25); other mesenchymal cell types, such as osteocytes (26, 27) and adipocytes (28), also express large amounts of RANKL supporting osteoclast differentiation and function. Using CellChat, we identified CAR cells, osteoblasts, and osteocytes as potential sources of RANKL supplied to RANK-positive BMECs (29) (*SI Appendix*, Fig. S10 *A* and *B*). An in-depth analysis of the cell types expressing RANKL in bone tissues was performed using scRNA-seq, identifying 13 cell types including CAR cells, osteoblasts, and osteocytes (19, 20, 30, 31) (Fig. 5 *A* and *B* and *SI Appendix*, Fig. S11*A*). CAR cells are essential for hematopoietic stem cell maintenance; they serve as the stem cells that will become mature osteoblasts (32–34). This analysis revealed that a subset of CAR cells, osteoblasts, and mesenchymal progenitors expressed *Tnfsf11* encoded RANKL (Fig. 5*C* and *SI Appendix*, Fig. S11 *B* and *C*). Previous studies have reported that CAR cells are enriched near BMECs in both sinusoidal and arterial regions (21). Indeed, some CXCL12-EGFP cells were observed in close proximity to the blood vessels in the bone marrow (Fig. 5*D*). Quantitative real-time PCR analyses revealed increased RANKL expression under osteoporotic conditions in CAR cells but not osteoblasts (Fig. 5 *E* and *F*).

### RANKL from Perivascular Stromal Cells Regulates Vascular Permeability in the Bone Marrow.

**Fig. 6.**
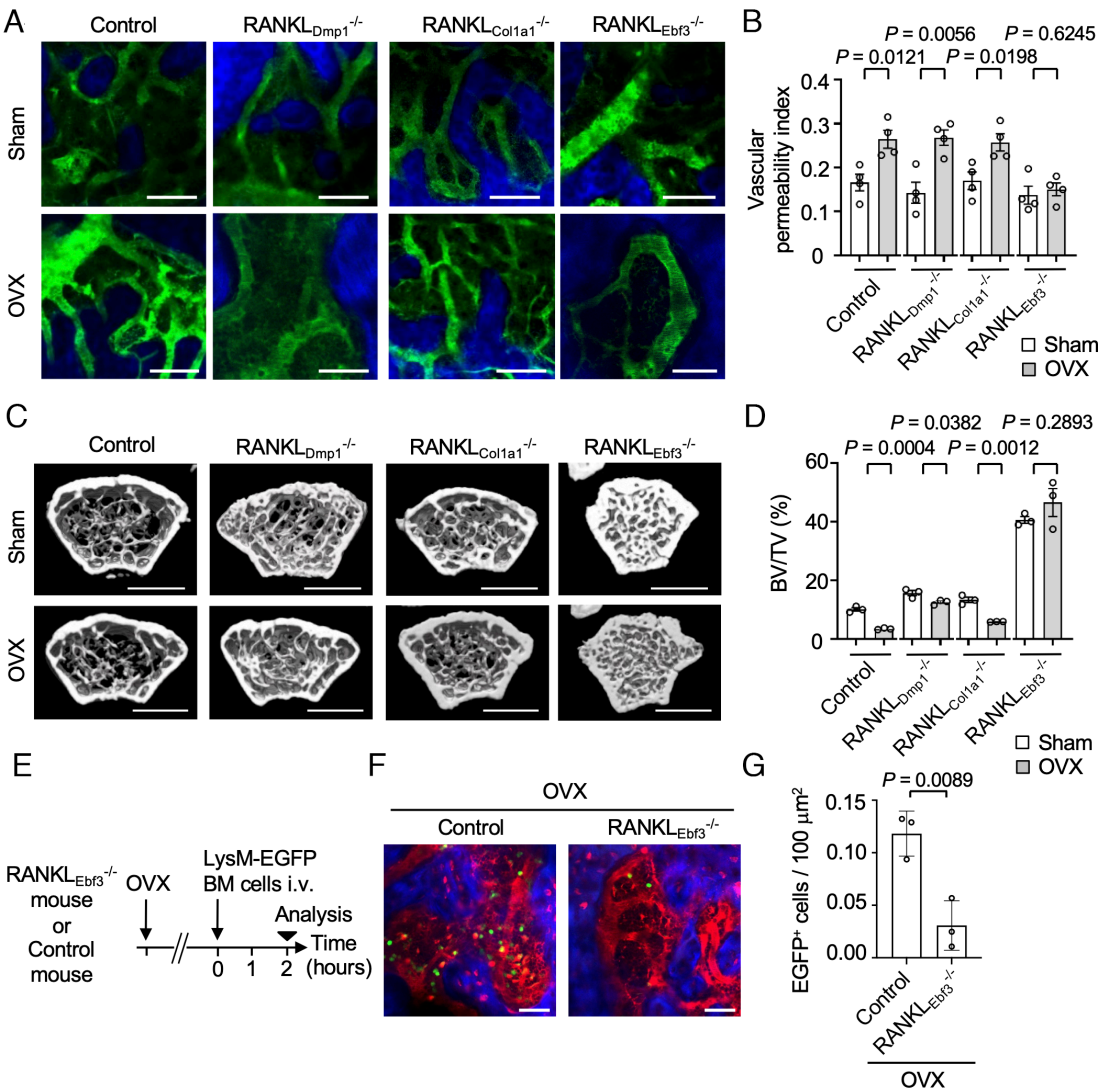
RANKL from perivascular stromal cells regulates vascular permeability in the bone marrow. (*A*) Representative intravital multiphoton images of calvarial bones of OVX-induced osteoporosis model (*Lower*) or sham-operated mice (*Upper*) in control (RANKL^f/f^), osteocyte-specific RANKL knockout (RANKL_Dmp1_^−/−^: *Dmp1*-Cre × RANKL^f/f^), osteoblast-specific RANKL conditional knockout (RANKL_Col1a1_^−/−^: *Col1a1*-CreERT2 × RANKL^f/f^), and CAR cell-specific RANKL conditional knockout mice (RANKL_Ebf3_^−/−^: *Ebf3*-CreERT2 × RANKL^f/f^). Blood vessels are shown in green, and bones are shown in blue. (Scale bar: 50 μm.) (*B*) Summary of the vascular permeability index in (*A*) (n = 4 biological replicates per group). (*C*) Representative micro-computed tomography images of the bone tissue from Control, RANKL_Dmp1_^−/−^, RANKL_Col1a1_^−/−^, and RANKL_Ebf3_^−/−^ mice before (*Upper*) and 1 mo after (*Lower*) OVX. (Scale bar: 1 mm.) (*D*) Summary of the BV/TV ratio determined from images in (*C*) (n = 3 biological replicates per group). (*E*) Experimental design. RANKL_Ebf3_^−/−^ or control mice 1 mo after OVX were injected with bone marrow cells isolated from LysM EGFP mice, and the transmigration frequency of EGFP-positive cells into sinusoidal capillaries of the bone marrow space was evaluated after 2 h. (*F*) Representative images of intravital multiphoton imaging of bone tissues in RANKL_Ebf3_^−/−^ or control mice injected with 70 kDa Texas Red-conjugated dextran after OVX (n = 3 biological replicates per group). Blood vessels are shown in red, bones are shown in blue, and EGFP-positive cells are shown in green. (Scale bar: 50 μm.) (*G*) Summary of the number of EGFP-positive cells in the bone marrow cavity area. Data are presented as the mean ± SEM. Statistical significance was determined by a two-tailed unpaired *t* test in *(B*, *D*, and *G*).

To identify the cell type responsible for RANKL-mediated regulation of vascular permeability, conditional KO mice exhibiting RANKL deletion in osteoblasts, osteocytes, and CAR stromal cells were generated by crossing RANKL-floxed mice (35), with collagen type 1 α1 (*Col1a1*)-CreERT2 (RANKL_Col1a1_^−/−^: *Col1a1*-CreERT2 × RANKL^f/f^) (36), dentin matrix acidic phosphoprotein 1 (*Dmp1*)-Cre (RANKL_Dmp1_^−/−^: *Dmp1*-Cre × RANKL^f/f^) (37), and early B cell factor 3 (*Ebf3*)-CreERT2 (RANKL_Ebf3_^−/−^: *Ebf3*-CreERT2 × RANKL^f/f^) (34) mice, respectively. The transcription factor EBF3 is preferentially expressed in CAR cells, and EBF3-expressing cells are self-renewing stem cells in adult bone marrow. Analysis of bone marrow vascular permeability in the conditional KO mice showed that, under osteoporotic conditions, vascular permeability was significantly greater in the RANKL_Dmp1_^−/−^ and RANKL_Col1a1_^−/−^ mice than in control mice (Fig. 6 *A* and *B*). In contrast, there was no increase in vascular permeability in the RANKL_Ebf3_^−/−^ mice, even under osteoporotic conditions (Fig. 6 *A* and *B*). Furthermore, BMD was significantly lower in the RANKL_Dmp1_^−/−^ or the RANKL_Col1a1_^−/−^ osteoporotic mice, whereas it was not significantly altered in the RANKL_Ebf3_^−/−^ mice (Fig. 6 *C* and *D*). Among nonovariectomized mice, highest bone density developed in the RANKL_Ebf3_^−/−^ mice than in the RANKL_Dmp1_^−/−^ or RANKL_Col1a1_^−/−^ mice (Fig. 6 *C* and *D*). Finally, we evaluated the migration of osteoclast precursor lineage cells from blood vessels into the bone marrow cavity in the RANKL_Ebf3_^−/−^ mice. The enhanced cellular migration, observed following OVX, was reduced in RANKL_Ebf3_^−/−^ mice (Fig. 6 *E*–*G*). These results indicated that CAR stromal cells constitute the major source of RANKL regulating both vascular permeability and bone homeostasis in vivo.

## Discussion

RANKL is a multifunctional cytokine, and since its identification as an essential factor for osteoclast differentiation and bone metabolism, RANKL has shown to be a role in broader fields of biomedical sciences. In addition to its originally identified roles in the skeletal and immune systems, lymph node organogenesis, and mammary gland development (38, 39), pleiotropic functions of RANKL have recently been reported, including in cancer metastasis (40, 41) and the central nervous system (42, 43). Furthermore, it has been suggested that RANKL increases vascular permeability of the skin and retina in an NO-dependent manner (44). However, whether RANKL regulates vascular permeability in the bone marrow, and how bone marrow vascular permeability affects bone metabolism has remained unclear. In this study, we established an imaging-based method for precisely and quantitatively evaluating vascular permeability and transluminal cellular migration in vivo. By means of this method, we could detect high vascular permeability and spontaneous transmigration of monocytoid cells in sinusoidal capillary of bone marrow spaces, which are augmented by RANKL.

RANKL is expressed in osteoblasts and osteocytes, where it serves as an essential factor for osteoclast differentiation (45). In this study, we found that CAR cells also produce substantial amount of RANKL. Specific deletion of RANKL expression in CAR cells could abrogate the vascular leakage, suggesting that CAR stromal RANKL is responsible for controlling permeability. Furthermore, CAR cells localize in the perivascular region and near the bone surface (32). BMD was higher in CAR cell-specific RANKL-KO mice than in RANKL-floxed mice. This result suggests the involvement of CAR cell-derived RANKL in controlling both vascular permeability and osteoclastogenesis. These results are consistent with previous reports on marrow adipogenic lineage precursors (MALPs), a population of mesenchymal progenitor cells committed to the adipocyte lineage. MALPs have been shown to express RANKL and promote osteoclast formation (46, 47). Notably, mice lacking MALP-derived RANKL exhibit increased bone mass and resistance to bone loss after OVX (46). Together, these results indicate that RANKL produced by CAR cells, including MALPs, not only enhances bone resorption but also regulates vascular permeability in the bone marrow microenvironment.

The bone marrow vasculature is divided into sinusoids, which consist of a single layer of endothelial cells and smooth muscle-invested arterioles (1, 19). Recently, single-cell analysis has revealed the diversity and specialization of BMECs (21, 48). In this study, we identified RANK-positive BMECs that control vascular permeability and spontaneous transmigration of monocytoid cells. We reanalyzed scRNA-seq datasets in which BMECs from the diaphysis, metaphysis, and epiphysis were individually examined (21). Our results show that RANK-positive BMECs are localized in metaphysis and diaphysis, but in epiphysis (*SI Appendix*, Fig. S12). In addition, we reanalyzed the data (48) and found that RANK-positive BMECs are influenced by age (*SI Appendix*, Fig. S13). These results suggest that RANK-positive BMEC is a definite subset involved in the regulation of bone metabolism.

RANK-positive BMECs control vascular permeability by regulating the expression patterns of adhesion molecules such as ICAM1 and VCAM1.The binding of RANKL to RANK leads to the recruitment of TRAF6, followed by the activation and nuclear translocation of NF-κB (49, 50). In vascular endothelial cells, NF-κB regulates VCAM1 and ICAM1 expression (51–54). In addition, RANK/RANKL signal may enhance transmigration of monocytoid cells by changing the expression of some tight junction molecules. Taken together, these results suggest a function for the RANK/RANKL axis in the bone marrow; it serves as an enhancer of the transluminal migration of osteoclast precursors into the bone marrow cavity by increasing the expression of adhesion molecules and thus promoting vascular permeability.

In this study, we demonstrated the importance of this regulatory function under pathological conditions. In osteoporosis, enhanced recruitment of circulating osteoclast precursor monocytes into the bone marrow cavity leads to an increase in the number of differentiated mature osteoclasts (18). RANKL has also been implicated in oncogenesis and metastasis; it contributes to bone metastasis by promoting tumor cell chemotactic activity (40, 41). A recent human study showed that high levels of serum RANKL are associated with the presence of disseminated tumor cells in the bone marrow of breast cancer patients (55). Mechanistically, RANKL produced by tumor cells and osteoblasts promotes osteoclastogenesis and bone resorption, while tumor cells further interact with bone-resident cells to amplify this process. Clinical and preclinical studies have shown that anti-RANKL therapy (e.g., denosumab) reduces bone metastases and tumor burden (56, 57). Additionally, adhesion molecules such as VCAM-1 and ICAM-1 contribute to metastatic progression by mediating tumor–bone interactions (58, 59). Given that bone metastases commonly occur via hematogenous spread to vascular-rich bones, our findings suggest that RANKL may also facilitate tumor cell extravasation into bone tissues by modulating the vascular permeability. These results highlight the potential of RANKL-targeting therapies to suppress both bone destruction and metastatic dissemination to bone.

**Fig. 7.**
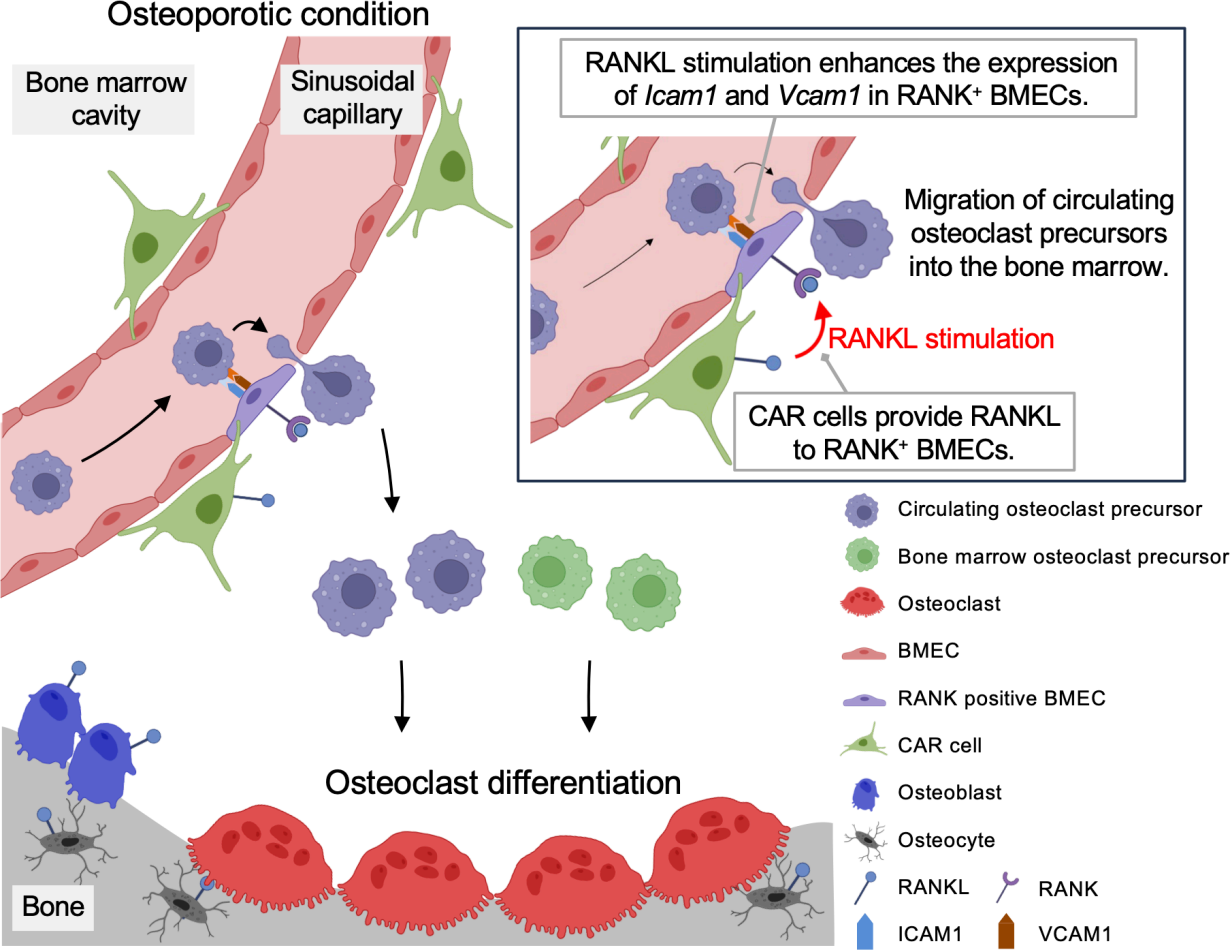
Schematic of the proposed regulatory mechanism of vascular permeability and osteoclastogenesis in the bone marrow. RANK positive BMECs have a role as a gatekeeper of blood vessels. RANKL from perivascular CAR cells promoted bone marrow vascular permeability and mobilized osteoclast precursors. In osteoporosis, the enhanced recruitment of circulating osteoclast precursors into the bone marrow leads to an increase in mature osteoclasts.

Overall, this study demonstrates the enhanced transluminal migration of osteoclast precursors into the bone marrow cavity under the direction of the RANK/RANKL axis through increased expression of adhesion molecules in the bone marrow. These events may represent a critical point in the regulation of bone homeostasis (Fig. 7).

## Materials and Methods

### Mice.

Female mice (8 to 12 wk old) and expressing LysM-EGFP (16) were used in this study, along with *Tnfsf11*-floxed (35), *Col1a1*-CreERT2 (36), *Dmp1*-Cre (37), *Ebf3*-CreERT2 (34), *Tnfrsf11a*-floxed (60), *Cdh5*-CreERT2;*Vcam1*-floxed (61), TRAP-tdTomato (8, 9), and *Cdh5*-CreERT2 (23) mice. CreERT2-mediated recombination was induced by intraperitoneally injecting 10- to 12-wk-old *Ebf3*-CreERT2; *Tnfsf11*^f/f^ mice with 2 mg of tamoxifen (Sigma), eight times every other day, *Col1a1*-CreERT2; *Tnfsf11*^f/f^ mice with 2 mg of tamoxifen, daily for 4 d, and *Cdh5*-CreERT2;*Vcam1*-floxed mice with 1 mg of tamoxifen, daily for 5 d. C57BL/6 WT mice were purchased from CLEA Japan, Inc. All mice were housed at a maximum of three animals per cage and randomly selected for experiments. The mice were fed a normal diet (MF diet; Oriental Yeast Co., Ltd.) and maintained at 23 ± 1.5 °C, 45 ± 15% relative humidity under a 12 h/12 h light/dark cycle in specific pathogen-free animal facilities at the University of Osaka. All animal experiments were approved by the Institutional Animal Experimental Committee of the University of Osaka.

### Multiphoton Intravital Bone Tissue Imaging.

Intravital microscopy of mouse calvaria was performed using a protocol modified from a previous study (8). Mice were anesthetized using isoflurane (Escain; 2.0% vaporized in 100% oxygen). The frontoparietal region of the skull was exposed, and the internal surfaces of bones adjacent to the bone marrow cavity were observed using a multiphoton excitation microscopy. The imaging systems consisted of a Nikon upright multiphoton microscope (A1R-MP) equipped with ×25 water-immersion objective (APO, N.A. 1.1), and a Carl Zeiss upright multiphoton microscope (LSM 780 NLO) equipped with a ×20 water-immersion objective (W Plan-Apochromat, N.A. 1.0). Both systems were driven by a laser (Chameleon Vision II Ti:Sapphire; Coherent, Inc.).

Intravital bone imaging in WT mice was performed using a Nikon multiphoton microscope. Blood vessels were visualized by intravenously injecting FITC-conjugated dextran of different molecular masses (40, 70, and 2,000 kDa; Sigma-Aldrich). Fluorescence images were acquired using external nondescanned detectors equipped with a band-pass emission filter at 500/50 nm (for FITC). The excitation wavelength was 880 nm.

Single cell suspensions were prepared from bone marrow cells isolated from the femur and tibia of LysM-EGFP mice by flushing. Mice were then intravenously injected with 2 × 10^7^ of these cells. After 2 h, LysM-positive cells in the bone marrow cavity were observed by using a Nikon multiphoton microscope. Blood vessels were visualized by intravenously injecting 70 kDa Texas Red-conjugated dextran (Sigma-Aldrich).

Some experiments in LysM-EGFP mice were performed using a Zeiss multiphoton microscope, and spectral images were acquired using specialized internal multiphotomultiplier detectors. The acquired raw images were subjected to spectral unmixing using ZEN software (Carl Zeiss) to create unmixed images that excluded autofluorescence. An excitation wavelength of 880 nm was used. Image drifts were corrected using Nikon Imaging Software (NIS-Elements) image analysis software (Nikon), in accordance with a standard protocol.

### Multiphoton Intravital Skin Imaging.

The methods for multiphoton intravital skin imaging are described in the *SI Appendix*.

### Drug Treatment.

To establish the RANKL-based osteoporosis model, GST-RANKL (Oriental Yeast) (1 mg/kg) dissolved in phosphate-buffered saline (PBS) was intraperitoneally injected into WT mice 2 d before imaging (15). To establish the postmenopausal osteoporosis model, mice were ovariectomized 1 mo prior to imaging.

WT mice were intraperitoneally injected with 0.2 mg of anti-VCAM-1 neutralizing antibody (BE0027, Bioxcell) and 0.2 mg of anti-ICAM-1 neutralizing antibody (BE0020-1, Bioxcell) or vehicle at 24-h intervals for 2 d before imaging.

### Image Data Analysis of the Vascular Permeability Index and Cell Migration.

Vascular permeability in the bone marrow was quantified by selecting three regions of interest in blood vessels and the bone marrow cavity (interstitial space) from the raw imaging data, then measuring their mean fluorescence intensities 30 min after the intravenous injection of FITC-conjugated dextran as described previously (62). The permeability index, defined as the ratio of the mean fluorescence intensity in the bone marrow cavities (interstitial space) to the mean fluorescence intensity in the blood vessel lumens, was calculated using NIS-Elements image analysis software (Nikon) (Fig. 1*B*).

Vascular permeability in the skin was quantified by selecting three regions of interest in blood vessels and the interstitial space from the raw imaging data, then measuring their mean fluorescence intensities 30 min after the intravenous injection of FITC-conjugated dextran. The permeability index, defined as the ratio of the mean fluorescence intensity in blood vessels to the mean fluorescence intensity in the interstitial space, was calculated using NIS-Elements image analysis software (Nikon).

Cell migration observed in intravital bone tissue imaging was quantitatively evaluated by dividing the number of LysM-positive cells in the bone marrow cavity by the area of the bone marrow cavity using NIS-Elements and IMARIS.

### Isolation of CAR Cells.

Bone marrow CAR cells were then isolated by flushing femurs and then enzymatically treated with 3 mg/mL collagenase type I (Worthington) and 100 μg/mL DNase I (Roche Diagnostics GmbH) for 30 min at 37 °C. CAR cells were isolated as Sca1^−^ CD45^−^ Ter119^−^ CD31^−^ CXCL12-EGFP^hi^ fraction (32, 33). Cells were sorted using an SH800 (Sony).

### Isolation of Osteoblasts.

Bone cells were isolated from mice using a method previously described (63). Hips, tibia, and femur bones were crushed using a mortar; the bone fragments were washed with Hank’s balanced salt solution (HBSS) (+) (Nakarai). These bone fragments were then incubated in a 3 mg/mL collagenase type I (Worthington solution in α-MEM for 25 min at 37 °C. The collagenase treatment was repeated two times (digestions 1 to 3). Next, the bone fragments were incubated in 5 mM ethylenediaminetetraacetic acid solution in magnesium- and calcium-free Dulbecco’s Phosphatase-Buffered Solutions (D-PBS) (Nakarai) with 1% bovine serum albumin (Sigma-Aldrich) for 25 min at 37 °C (digestion 4). The bone tips were washed with HBSS (+) and treated with collagenase again (digestion 5). Osteoblasts were isolated as Lin^−^ CD31^−^ Sca1^−^ CD51^+^ fraction from digestions 1 to 5. Cells were sorted using SH800 (Sony).

### Cell Transplantation.

Single-cell suspensions were prepared using bone marrow cells isolated from LysM-EGFP mouse femurs by flushing. WT mice were then intravenously injected with 1 × 10^7^ of these cells. After 2 h, bone marrow cells from the femurs were isolated by flow cytometry (SH800; Sony) and analyzed using FlowJo software (Tree Star).

### Bone Morphometry.

Femurs excised from mice were fixed overnight in 4% (v/v) paraformaldehyde. The isolated bone samples were then imaged using a cone-beam X-ray micro-computed tomography system (ScanXmate-RB090SS150; Comscantecno). Three-dimensional reconstruction images were generated and analyzed using TRI/3D-BON software (RATOC System Engineering).

The left femurs were extracted from RANK_Cdh5_^−/−^ mice and performed bone morphometric analysis as described previously (12).

### TEER in Human Dermal Microvascular Endothelial Cells (HDMECs).

HDMECs (Lonza, 4 × 104 cells) were seeded in cell culture inserts with a pore size of 0.4 µm (BD Falcon). After 96 h, RANKL (10 μg/mL) or an equivalent volume of PBS (control) was added to the bottom chamber of the culture device. TEER was measured using the CellZscope system (NanoAnalytics) at 6 h after treatment (64). The TEER values (Ω/cm^2^) of RANKL-treated or PBS-treated cells were normalized to the values at time (t) = 0.

### qRT-PCR.

qRT-PCR was performed using the Thermal Cycler Dice Real-Time System TP800 (Takara) and the following specific primer pairs (forward and reverse, respectively): *Tnfsf11* (5′-CAGCATCGCTCTGTTCCTGTA-3′ and 5′-CTGCGTTTTCATGGAGTCTCA-3′), *Tnfrsf11a* (5′-CTGCTCCTCTTCATCTCTGTG-3′ and 5′-CTTCTGGAACCATCTTCTCCTC3′), and *Gapdh* (5′-ACCACAGTCCATGCCATCAC-3′ and 5′-TCCACCACCCTGTTGCTGTA-3′).

### scRNA-seq.

The methods for scRNA-seq are described in the *SI Appendix*.

### Statistical Analysis.

Data were analyzed using GraphPad Prism ver. 9 (GraphPad Software, Inc.) and are presented as the mean ± SEM. Statistical analyses were performed using two-tailed unpaired *t* tests or Wilcoxon rank sum tests for comparisons between two groups, and one-way ANOVA with Tukey’s test for comparisons among three or more groups. A *P*-value < 0.05 was considered statistically significant. The numbers of samples and mice are indicated in the figure legends. The results are representative of at least three independent experiments, unless otherwise indicated. Biological replicates comprised samples from different mice. The required sample sizes were estimated by considering variations and means. Attempts were made to reach reliable conclusions using sample sizes that were as small as possible.

## Supplementary Material

Appendix 01 (PDF)

Movie S1. **Intravital imaging of high vascular leakage in the bone marrow**. Representative intravital multiphoton images of bone tissues from wild-type mice injected with fluorescein isothiocyanate-conjugated dextran [70 kDa (left panel) and 2,000 kDa (right panel)]. Blood vessels are shown in green, and bones are shown in blue. Scale bar: 50 μm. Playback speed: 150×.

Movie S2. **Intravital imaging of the continuous entrance of monocytoid cells into the bone marrow**. Representative intravital multiphoton images of bone tissues from control (left panel) and RANKL-induced osteoporotic (right panel) LysM-EGFP mice injected with Alexa Fluor 594-conjugated isolectin IB4. Blood vessel walls are shown in red, and EGFP-positive cells are shown in green. Scale bar: 10 μm. Playback speed: 30×.

## Data Availability

The scRNA-seq data have been deposited in the ArrayExpress database at EMBL-EBI under accession number E-MTAB-15929 (65). All other data are included in the article and/or supporting information.
