# Supplementary material for: The RANK/RANKL axis controls vascular dynamics in the bone marrow
Source: Proc Natl Acad Sci U S A. 2025 Nov 3;122(45):e2425366122. doi: 10.1073/pnas.2425366122 (PMC12625855; doi:10.1073/pnas.2425366122)
Supplement: Supplementary file 1 — Appendix 01 (PDF) [file pnas.2425366122.sapp.pdf]

PNAS

Supporting Information for
The RANK/RANKL axis controls vascular dynamics in the bone marrow

Takeshi Kaneko [a,b,c,1], Shinya Yari [a,1], Junichi Kikuta [a,c,d,2,3], Yoshiki Omatsu [c,e], Shigeto Seno [f], Sumire Kikuchi [a,c], Kazuma Sato [a,c], Kentaro Fujii [a,c], Takao Sudo [a], Tetsuo Hasegawa [a], Kunimaro Furuta [g], Qianqian Guo [g], Samar H Ibrahim [g], Kosuke Muraoka [h], Yoshiaki Okada [h], Yoshiaki Kubota [i], Daisuke Okuzaki [c,j], Yasuhiro Kobayashi [k], Atsushi Kumanogoh [b,c], Nobuyuki Udagawa [k], Takashi Nagasawa [c,e], Josef M. Penninger [l,m,n], and Masaru Ishii [a,c,d,o,2]

[a] Department of Immunology and Cell Biology, Graduate School of Medicine and Frontier Biosciences, The University of Osaka, Osaka, Japan.
[b] Department of Respiratory Medicine and Clinical Immunology, Graduate School of Medicine, The University of Osaka, Osaka, Japan.
[c] WPI-Immunology Frontier Research Center, The University of Osaka, Osaka, Japan.
[d] Laboratory of Bioimaging and Drug Discovery, National Institutes of Biomedical Innovation, Health and Nutrition, Osaka, Japan.
[e] Laboratory of Stem Cell Biology and Developmental Immunology, Graduate School of Medicine and Frontier Biosciences, The University of Osaka, Osaka, Japan.
[f] Department of Bioinformatic Engineering, Graduate School of Information Science and Technology, The University of Osaka, Osaka, Japan.
[g] Division of Gastroenterology and Hepatology, Mayo Clinic, Rochester, Minnesota, USA.
[h] Graduate School of Pharmaceutical Sciences, The University of Osaka, Osaka, Japan.
[i] Department of Anatomy, Keio University School of Medicine, Tokyo, Japan.
[j] Genome Information Research Center, Research Institute for Microbial Diseases, The. University of Osaka, Osaka, Japan.
[k] Department of Biochemistry, Matsumoto Dental University, Nagano, Japan.
[l] Helmholtz Centre for Infection Research, Braunschweig, Germany.
[m] Department of Laboratory Medicine, Medical University of Vienna, Vienna, Austria.
[n] Department of Medical Genetics, Life Sciences Institute, University of British Columbia, Vancouver, Canada.
[o] Life-omics Research Division, Institute for Open and Transdisciplinary Research Initiative, The University of Osaka, Osaka, Japan.

[1] T.K and S.Y contributed equally to this work.
[2] To whom correspondence may be addressed. Email: mishii@icb.med.osaka-u.ac.jp or jkikuta@icb.med.osaka-u.ac.jp
[3] Present address: Division of Immunology, Department of Future Medical Sciences, Graduate School of Medicine, Kobe University, Hyogo, Japan.

Corresponding Author information.
Masaru Ishii, Department of Immunology and Cell Biology, Graduate School of. Medicine and Frontier Biosciences, Osaka University, 2-2 Yamada-oka, Suita, Osaka 565-0871, Japan
Tel: +81-6-6879-3881, Fax: +81-6-6879-3889
E-mail: mishii@icb.med.osaka-u.ac.jp

Junichi Kikuta, Department of Immunology and Cell Biology, Graduate School of. Medicine and Frontier Biosciences, Osaka University, 2-2 Yamada-oka, Suita, Osaka 565-0871, Japan
Tel: +81-6-6879-3881, Fax: +81-6-6879-3889
E-mail: jkikuta@icb.med.osaka-u.ac.jp

This PDF file includes:

Materials and Methods
Reference
Figures S1 to S13
Legends for Movies S1 to S2

Other supporting materials for this manuscript include the following:

Movies S1 to S2

Materials and Methods

Multiphoton intravital skin imaging

Intravital skin imaging in WT mice was performed using a protocol modified from a previous study (1). Mice were anesthetized using isoflurane (Escain; 2.0% vaporized in 100% oxygen). The ear lobes or skin of the back were exposed, and observed using a multiphoton excitation microscopy. The imaging systems consisted of a Nikon inverted multiphoton microscope (A1R-MP) equipped with ×20 multiimmersion objective (Plan Fluor, N.A. 0.75). The system was driven by a laser (Chameleon Vision II Ti:Sapphire; Coherent, Inc.). Blood vessels were visualized by intravenously injecting FITC-conjugated dextran of different molecular masses (40, 70, and 2,000 kDa; Sigma-Aldrich). Fluorescence images were acquired using external non-descanned detectors equipped with a band-pass emission filter at 500/50 nm (for FITC). The excitation wavelength was 880 nm. Image drifts were corrected using NIS-elements image analysis software (Nikon), in accordance with a standard protocol.

scRNA-seq of BMECs

Femur, tibia, and pelvis were collected from euthanized mice. After complete removal of the soft tissues, bones were crushed in Hank's balanced salt solution [(HBSS) (+)] using a cold mortar. Bone tips were incubated for 1 h at 37 °C in a solution containing 3 mg collagenase type 1 (Worthington)/mL, prepared in HBSS (+). The dissociated cells were collected and subjected to hemolysis in ammonium chloride potassium lysis buffer (A1049201; Gibco). Endothelial cells were isolated using flow cytometry and incubated for 15 min with CD16/32 antibody (553141; BD Biosciences). Next, they were stained for 30 min with anti-CD45-FITC (103108; BioLegend), anti-Ter119-FITC (557915; BD Biosciences), and anti-CD31-allophycocyanin (APC) (405207: BioLegend). $CD31^+$ $CD45^-$ $Ter119^-$ cells were isolated as BMECs using a SH800 cell sorter (Sony).

Single-cell suspensions were processed using the 10× Genomics Chromium Controller, in accordance with the protocol outlined in the Chromium Next GEM Chip K Single Cell Kit (cat.# PN-1000287) user guide. The Chromium Next GEM Single Cell 3' Library & Gel Bead Kit v1.1 (cat.# PN-1000128), Chromium Next GEM Chip G Single Cell Kit (cat.# PN-1000127), and Single Index Kit T Set A (cat.# PN-1000213) were used during this process. In accordance with the manufacturer's recommendations, ~16,500 live cells per sample were loaded onto the Chromium controller to generate 10,000 single-cell gel-bead emulsions for library preparation and sequencing. Oil droplets of encapsulated single cells and barcoded beads (GEMs) were subsequently reverse transcribed in a Veriti thermal cycler (Thermo Fisher Scientific), resulting in mRNA-derived cDNA tagged with a cell barcode and unique molecular index (UMI).

Next, cDNA was amplified to generate single-cell libraries, in accordance with the manufacturer's protocol. cDNA was quantified using an Agilent bioanalyzer high-sensitivity DNA assay (Agilent, High-Sensitivity DNA kit, cat.# 5067-4626). Subsequently, the amplified cDNA was enzymatically fragmented, end-repaired, and polyA-tagged. Cleanup and size selection of the amplified cDNA were performed using SPRIselect magnetic beads (Beckman-Coulter, SPRIselect, cat.# B23317). Illumina sequencing adapters were ligated to the size-selected fragments and cleaned with SPRIselect magnetic beads. Finally, sample indices were selected and amplified; double-sided size selection was performed using SPRIselect magnetic beads. The samples were sequenced in paired-end mode on a DNBSEQ-G400RS (MGI).

scRNA-seq of mesenchymal cells

The right auricles of euthanized mice were cut, and 10 mL of PBS were injected into the left ventricle for perfusion. Calvaria were isolated and washed with HBSS (+), cut into 1-mm sections, and incubated in a solution containing 3 mg type II collagenase (Worthington)/mL dissolved in HBSS (+) for 25 min at 37°C. The collagenase step was repeated two times (digestions 1-3). Next, the bone pieces were incubated in a 5 mM EDTA solution prepared in D-PBS containing 1% bovine serum albumin for 25 min at 37°C (digestion 4). The bone tips were washed with HBSS (+) and treated with collagenase, EDTA, and collagenase in this order (digestions 5-7). Digestions 1-7 were subjected to flow cytometry for cell isolation using a SH800 cell sorter (Sony). Isolated murine cells were blocked with anti-CD16/32 antibody (553141; BD Biosciences) for 15 min, then stained with anti-Ter119-biotin (116204; BioLegend) for 15 min and anti-CD45-

APC (103112; BioLegend), anti-CD31-APC (102409; BioLegend), and streptavidin-APC (405207; BioLegend) for 30 min. The cells were isolated as the $CD31^-$ $CD45^-$ $Ter119^-$ fraction from digestions 1-7 using a SH800 cell sorter (Sony).

Single-cell suspensions were processed using the 10× Genomics Chromium Controller, in accordance with the protocol outlined in the Chromium Next GEM Chip K Single Cell Kit (cat.# PN-1000287) user guide. The Chromium Next GEM Single Cell 5' Library & Gel Bead Kit v1.1 (cat.# PN-1000167), Chromium Next GEM Chip G Single Cell Kit (cat.# PN-1000127), and Single Index Kit T Set A (cat# PN-1000213) were used during this process. In accordance with the manufacturer's recommendations, approximately 16,500 live cells per sample were loaded onto the Chromium controller to generate 10,000 single-cell gel-bead emulsions for library preparation and sequencing. Oil droplets of encapsulated single cells and barcoded beads (GEMs) were subsequently reverse transcribed in a Veriti thermal cycler (Thermo Fisher Scientific), resulting in mRNA-derived cDNA tagged with a cell barcode and UMI. Next, cDNA was amplified to generate single-cell libraries, in accordance with the manufacturer's protocol. cDNA was quantified using an Agilent bioanalyzer high-sensitivity DNA assay (Agilent, High-Sensitivity DNA Kit, cat.# 5067-4626). Subsequently, the amplified cDNA was enzymatically fragmented, end-repaired, and polyA-tagged. Cleanup and size selection of the amplified cDNA were performed using SPRIselect magnetic beads (Beckman-Coulter, SPRIselect, cat.# B23317). Illumina sequencing adapters were ligated to the size-selected fragments and cleaned using SPRIselect magnetic beads. Finally, sample indices were selected and amplified; double-sided size selection was performed using SPRIselect magnetic beads. The samples were sequenced in paired-end mode on a DNBSEQ-G400RS (MGI).

scRNA-seq data analysis

The resulting raw reads were processed using cellranger 6.0.0 (10× Genomics) (51). Data analysis was conducted using R (version 4.2.0) and Seurat (version 5.0.3). In the scRNA-seq analysis of endothelial cells, cells were excluded if they had < 200 genes or > 4500 genes, and if they had > 5% mitochondrial genes. The data processing sequence included NormalizeData, ScaleData, FindVariableGenes, RunPCA, FindNighbors, and FindClusters. For comparative analysis between RANKL-treated and control groups, clusters with high expression of $\textit{Cdh5}^{high}$ and $\textit{Pecam1}^{high}$ were identified as endothelial cell subsets. These subsets from both groups were merged, normalized using the SCTransform function, and subjected to the RunHarmony function with the SCT assay for batch effect correction.

Cells were categorized based on the raw expression level of *Tnfrsf11*: RANK-positive cells had a level > 0, while RANK-negative cells had a level of 0. Differential expression analysis between the RANKL-treated and control groups was conducted using the FindAllMarkers function, which used the Wilcoxon rank-sum test to evaluate *P* values. $P < 0.05$ was defined as statistically significant. GO enrichment analysis was conducted using the compareCluster function, specifically choosing the "enrichGO" method (2). CellChat software, along with CellChatDB database, was used for cellular interaction analysis, following the methodology outlined in the original tutorial (3).

The scRNA-seq of mesenchymal cells was performed twice, and these datasets were integrated for analysis. Cells with < 1000 genes, > 4000 genes, or > 5% mitochondrial genes were excluded. Following integration, data processing included NormalizeData, FindVariableFetures, ScaleData, SCTransform, and RunPCA, followed by batch effect correction using the RunHarmony function with the SCT assay.

Reference

1. G. Egawa, S. Nakamizo, Y. Natsuaki, H. Doi, Y. Miyachi, K. Kabashima, Intravital analysis of vascular permeability in mice using two-photon microscopy. *Sci Rep.* **3**, 1932 (2013).
2. G. Yu, L. G. Wang, Y. Han, Q. Y. He, clusterProfiler: an R package for comparing biological themes among gene clusters. *Omics* **16**, 284–287 (2012).
3. S. Jin, C. F. Guerrero-Juarez, L. Zhang, I. Chang, R. Ramos, C.-H. Kuan, P. Myung, M. V. Plikus, Q. Nie, Inference and analysis of cell-cell communication using CellChat. *Nat. Commun.* **12**, 1088 (2021).
4. T. Iga, H. Kobayashi, D. Kusumoto, T. Sanosaka, N. Fujita, I. Tai-Nagara, T. Ando, T. Takahashi, K. Matsuo, K. Hozumi, K. Ito, M. Ema, T. Miyamoto, M. Matsumoto, M. Nakamura,

H. Okano, S. Shibata, J. Kohyama, K. K. Kim, K. Takubo, Y. Kubota, Spatial heterogeneity of bone marrow endothelial cells unveils a distinct subtype in the epiphysis. *Nat. Cell Biol.* **25**, 1415-1425 (2023).

5. V. Mohanakrishnan, K. K. Sivaraj, H. W. Jeong, E. Bovay, B. Dharmalingam, M. G. Bixel, V. V. Dinh, M. Petkova, I. P. Ugarte, Y. T. Kuo, M. Gurusamy, B. Raftrey, N. T. L. Chu, S. Das, P. E. R. Coronado, M. Stehling, L. Sävendahl, A. S. Chagin, T. Mäkinen, K. Red-Horse, R. H. Adams, Specialized post-arterial capillaries facilitate adult bone remodelling. *Nat. Cell Biol.* **26**, 2020-2034 (2024).

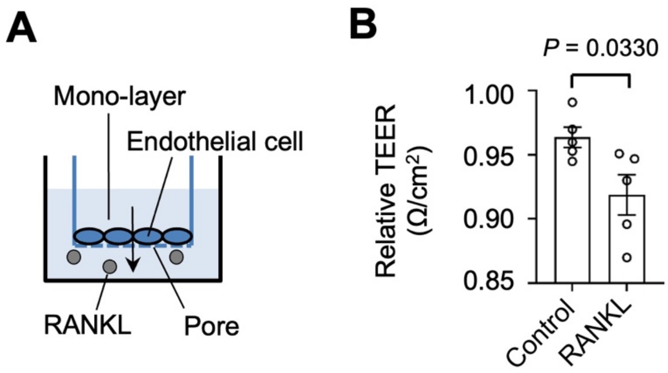

Fig. S1. Investigation of the directly effect of RANKL on endothelial cells by *in vitro* model. (**A**) Experimental design. The transendothelial electrical resistance of cultured microvascular endothelial cells treated with RANKL was evaluated in *in vitro* model. (**B**) The transendothelial electrical resistance of cultured microvascular endothelial cells decreased after RANKL treatment (n = 5 biological replicates per group). Data are presented as the mean ± SEM. Statistical significance was determined by a two-tailed unpaired *t*-test in (**B**).

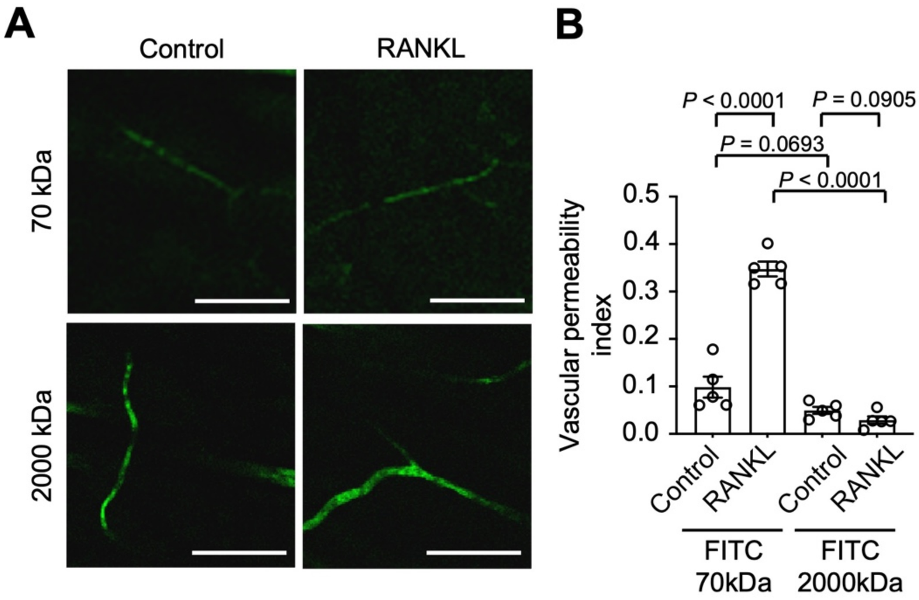

Fig. S2. RANKL enhanced vascular permeability in the skin.

(**A**) Representative intravital multiphoton images of skin from WT mice injected with fluorescein isothiocyanate (FITC)-conjugated dextran of different molecular masses (70 and 2,000 kDa) (n = 5 biological replicates per group). Blood vessels are shown in green. Collagen fibers are shown in blue. Scale bar: 100 µm. (**B**) Summary of the vascular permeability index in the skin (n = 5 biological replicates per group). Data are presented as the mean ± SEM. Statistical significance was determined by one-way analysis of variance (ANOVA) with Tukey's test in (B).

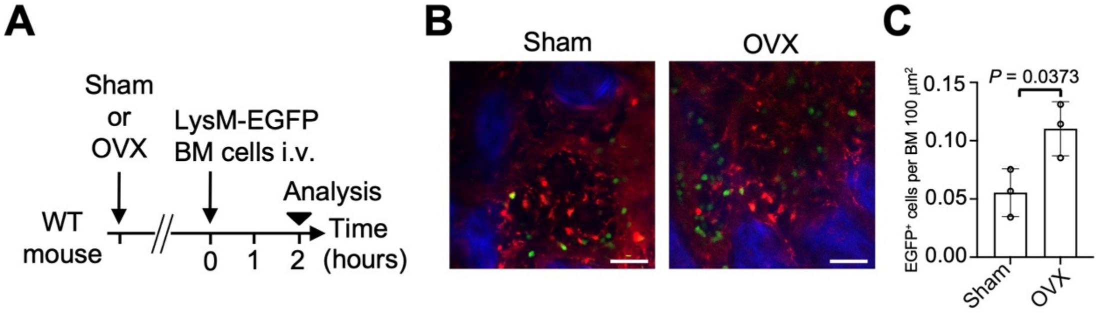

Fig. S3. OVX-dependent control of the recruitment of monocyte/macrophage lineage cells in the bone marrow.

(**A**) Experimental design. Sham-operated or ovariectomized (OVX) mice WT mice were injected with bone marrow cells isolated from LysM EGFP mice, and the transmigration frequency of EGFP-positive cells into sinusoidal capillaries of the bone marrow space was evaluated after 2 h. (**B**) Representative images of intravital multiphoton imaging of bone tissues in sham-operated mice (*left* panel) and OVX mice (*right* panel) injected with 70 kDa Texas Red-conjugated dextran. Blood vessels are shown in red, bones are shown in blue, and EGFP-positive cells are shown in green. Scale bar: 50 µm. (**C**) Summary of the number of EGFP-positive cells in the bone marrow cavity area. Data are presented as the mean ± SEM. Statistical significance was determined by a two-tailed unpaired *t*-test in (**C**).

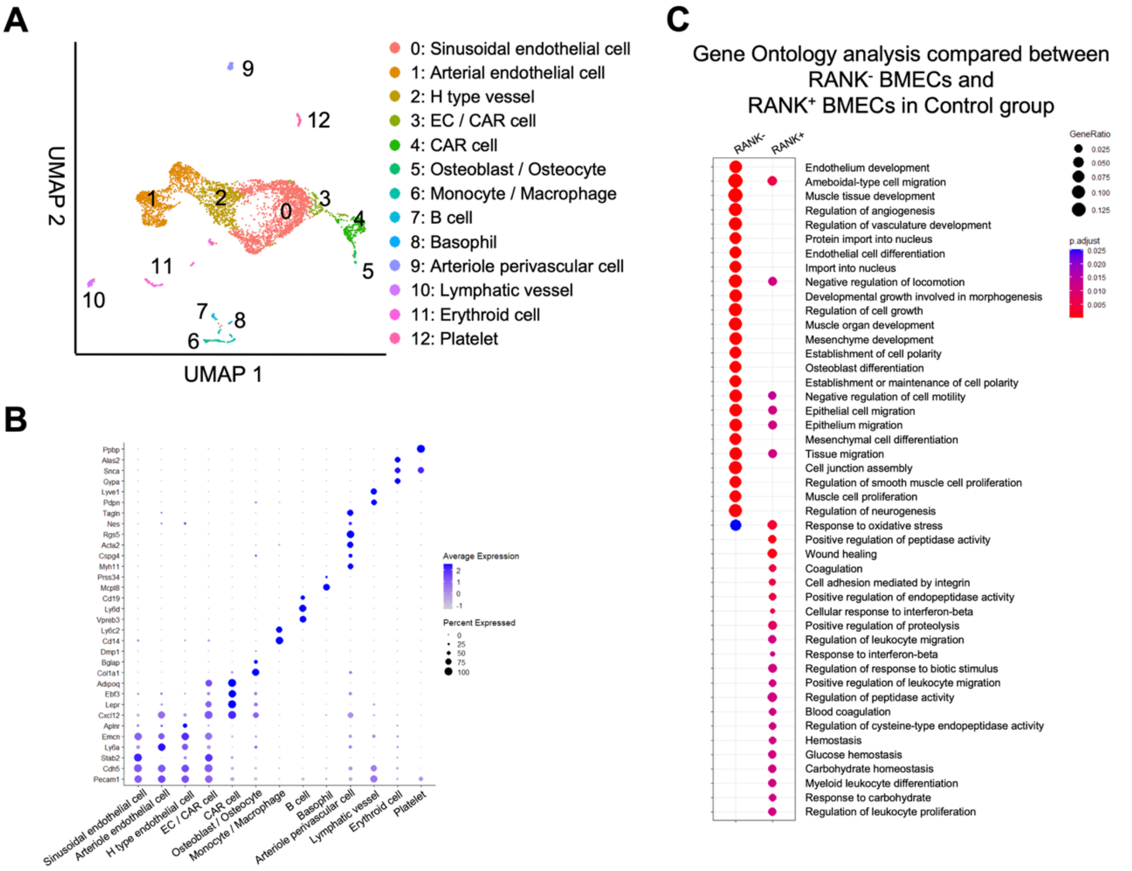

Fig. S4. scRNA-seq analysis of BMECs in control group.

(**A**) Clustering of scRNA-seq of BMECs in control group. The data were split into 12 clusters. (**B**) Dot pots showing the marker genes in each cell types. Cluster 0, 1, and 2 were defined as *Cdh5* and *Pecam1* positive BMECs. (**C**) Heatmap showing on difference of gene expression analysis of RANK negative (RANK$^-$) BMECs and RANK positive (RANK$^+$) in control group. Statistical significance was determined by the Wilcoxon rank-sum test. The *P* value of no more than 0.05 was defined as the significantly difference.

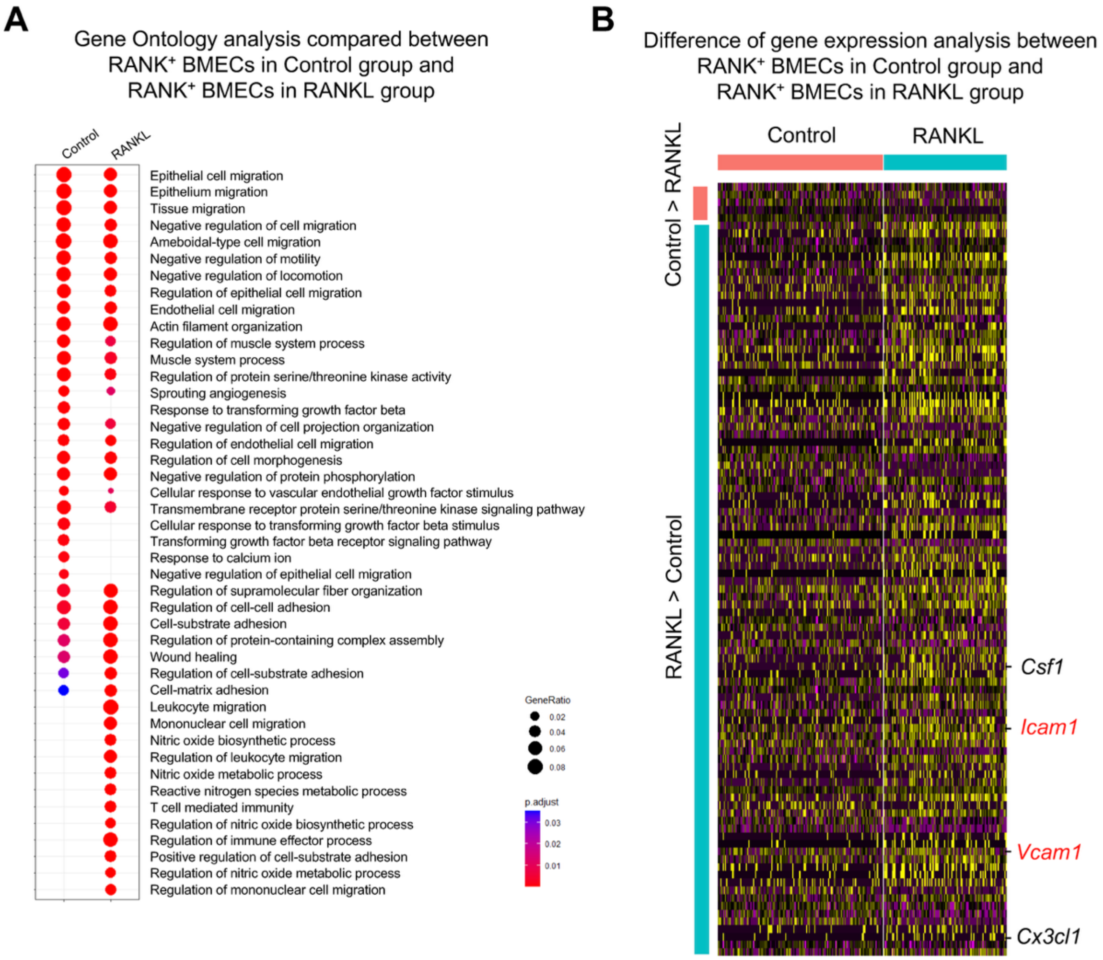

Fig. S5. The genetic effects in RANK⁺ BMECs induced by RANKL stimulation.

(**A**) Gene Ontology analysis of RANK positive (RANK⁺) BMECs in RANKL group and control group. (**B**) Heatmap showing on difference of gene expression analysis of RANK⁺ BMECs in control group and RANKL group. Statistical significance was determined by the Wilcoxon rank-sum test. The *P* value of no more than 0.05 was defined as the significantly difference.

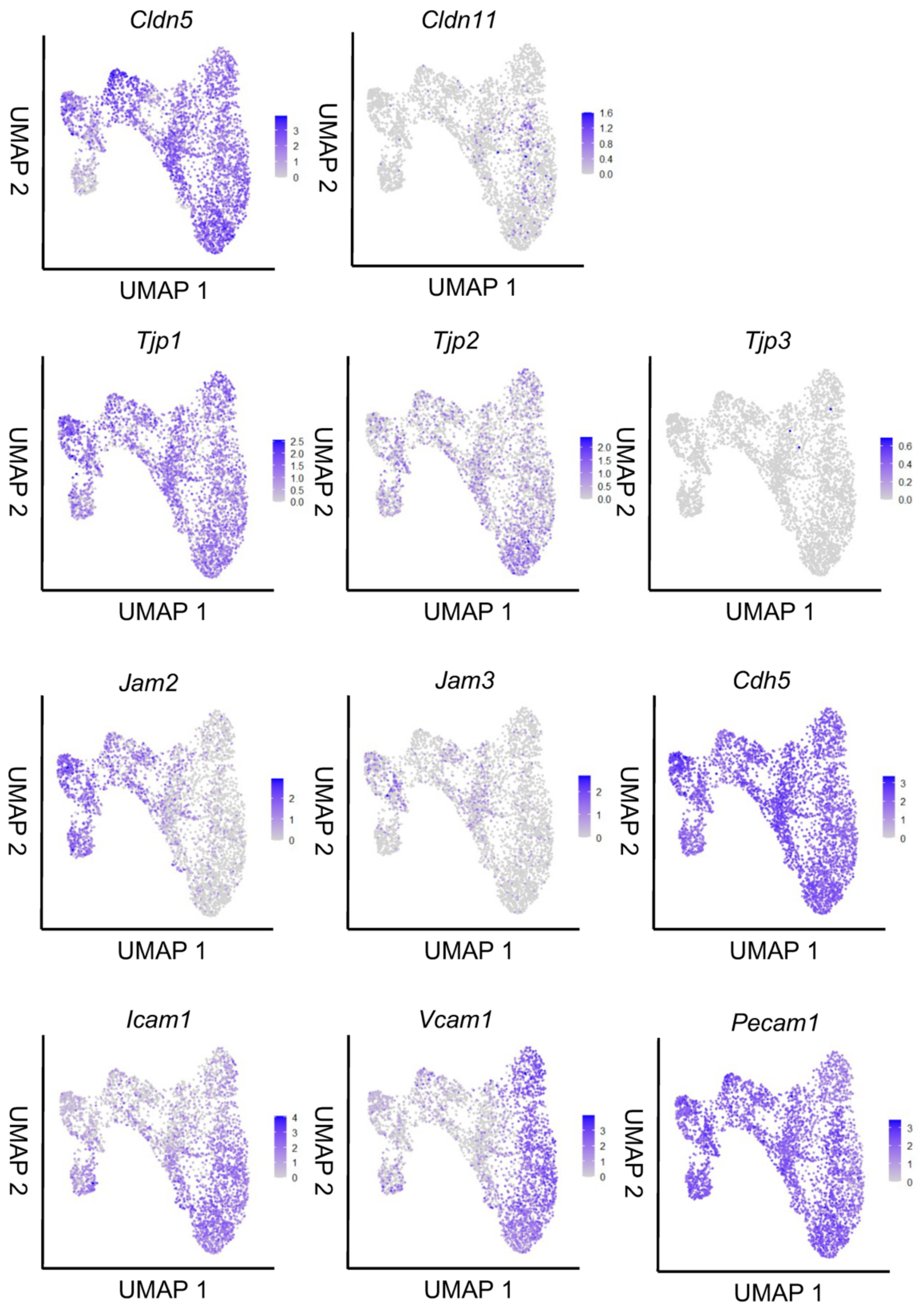

Fig. S6. Feature plots of the gene markers related to tight junctions, adherens junctions, and cell adhesion molecules in the bone marrow endothelial cells.

Feature plots of the gene markers related to tight junctions (*Cldn5, Cldn11, Tjp1, Tjp2, Tjp3, Jam2, Jam3*), adherens junctions (*Cdh5*), and cell adhesion molecules (*Icam1, Vcam1, Pecam1*) in the bone marrow endothelial cells.

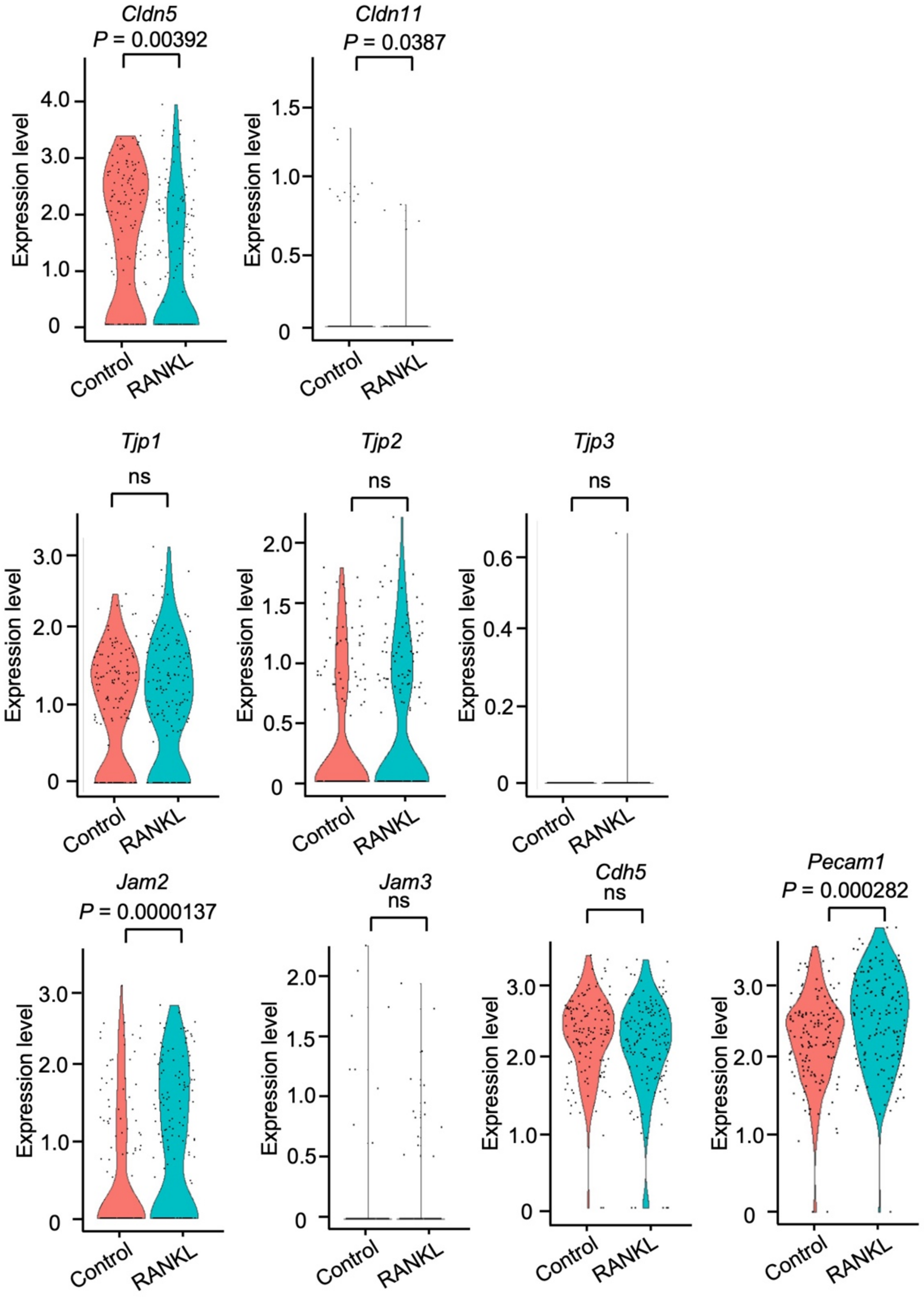

Fig. S7. Violin plots of gene expression related to tight junctions, adherens junctions, and cell adhesion molecule in RANK-positive bone marrow endothelial cells following RANKL treatment.

Violin plots of gene expression related to tight junctions (*Cldn5, Cldn11, Tjp1, Tjp2, Tjp3, Jam2, Jam3*), adherens junctions (*Cdh5*), and cell adhesion molecule (*Pecam1*) in RANK-positive bone marrow endothelial cells of control and RANKL treatment group.

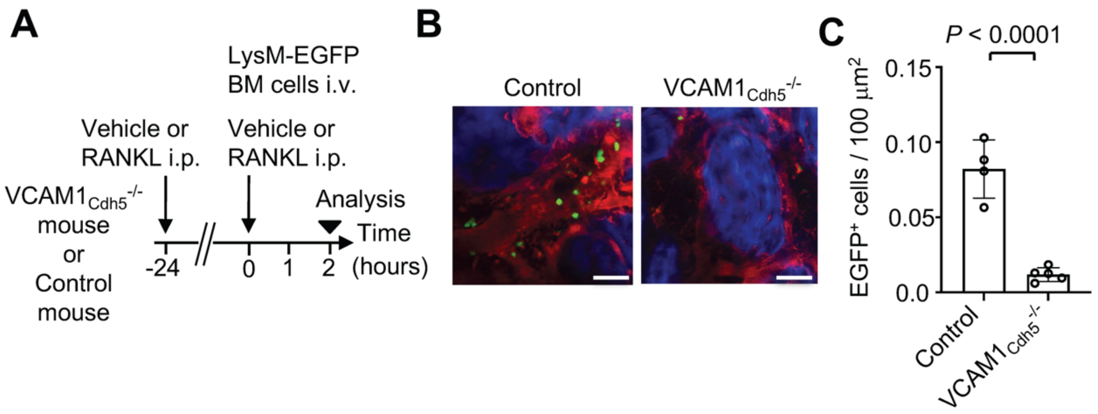

Fig. S8. Endothelial VCAM1-dependent control of the recruitment of monocyte/macrophage lineage cells in the bone marrow.

(**A**) Experimental design. Endothelial cell-specific VCAM-1 knockout mice ($VCAM1_{Cdh5}^{-/-}$: *Cdh5*-CreERT2×$VCAM1^{f/f}$) and control mice ($VCAM1^{f/f}$) were injected with bone marrow cells isolated from LysM-EGFP mice. The transmigration frequency of EGFP-positive cells into sinusoidal capillaries in the bone marrow was assessed 2 hours later. Soluble RANKL (2 mg/kg) or vehicle was administered intraperitoneally at 24-hour intervals for two consecutive days. (**B**) Representative images of intravital multiphoton imaging of bone tissues in $VCAM1_{Cdh5}^{-/-}$ or control mice injected with 70 kDa Texas Red-conjugated dextran. Blood vessels are shown in red, bones are shown in blue, and EGFP-positive cells are shown in green. Scale bar: 50 μm. (**C**) Summary of the number of EGFP-positive cells in the bone marrow cavity area. Data are presented as the mean ± SEM. Statistical significance was determined by a two-tailed unpaired *t*-test in (**C**).

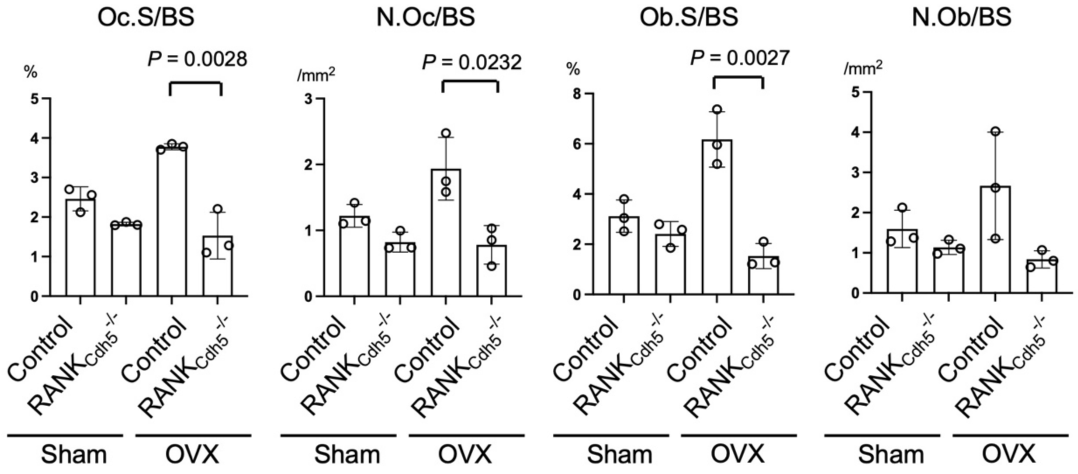

Fig. S9. Bone morphometric analysis of the femur in endothelial cell-specific RANK knockout mice.

Endothelial-specific RANK-deficient mice ($RANK_{Cdh5}^{-/-}$: *Cdh5*-CreERT2×$RANK^{f/f}$) and control mice ($RANK^{f/f}$) were ovariectomized or sham-operated. Femurs were collected, and bone morphometric analysis was performed to assess the osteoclast surface area (Oc.s/BS), osteoclast number (N.Oc/BS), osteoblast surface area (Ob.S/BS) and osteoblast number (N.Ob/BS).

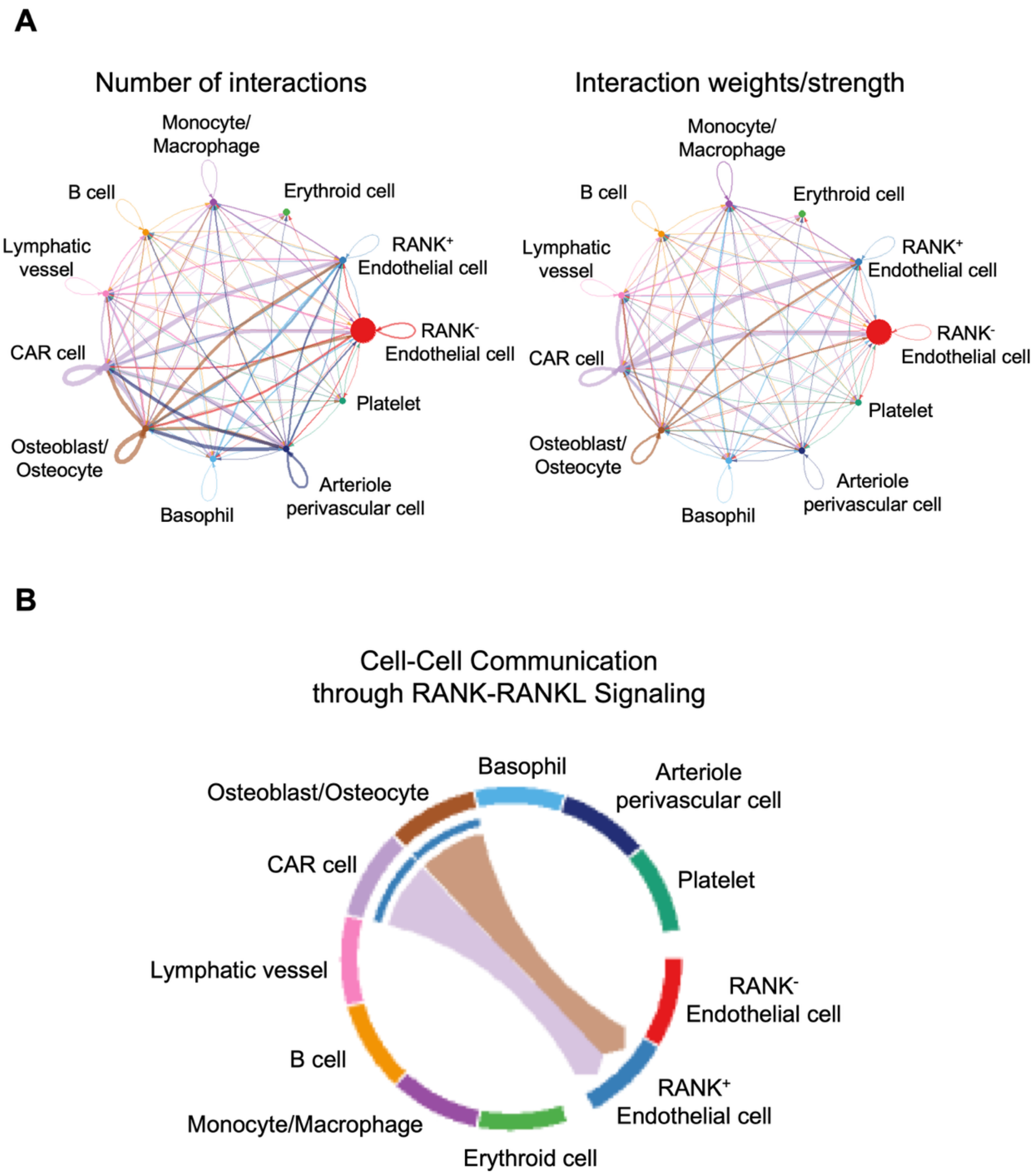

Fig. S10. The prediction of RANKL source to enhance bone marrow vascular permeability using CellChat.

(**A**, **B**) In the scRNA-seq data of Fig. S4 (**A**), the communication between cell types each other was predicted by CellChat. (**A**) Chord diagrams showing number (left) of interactions and interaction wights/strength (right). The line thickness the interaction number or weights between each cell types. (**B**) Chord diagrams showing the predicted cell-cell communication through RANK-RANKL signaling. Thickness of the lines shows to the weight of the predicted interaction by RANK-RANKL signaling.

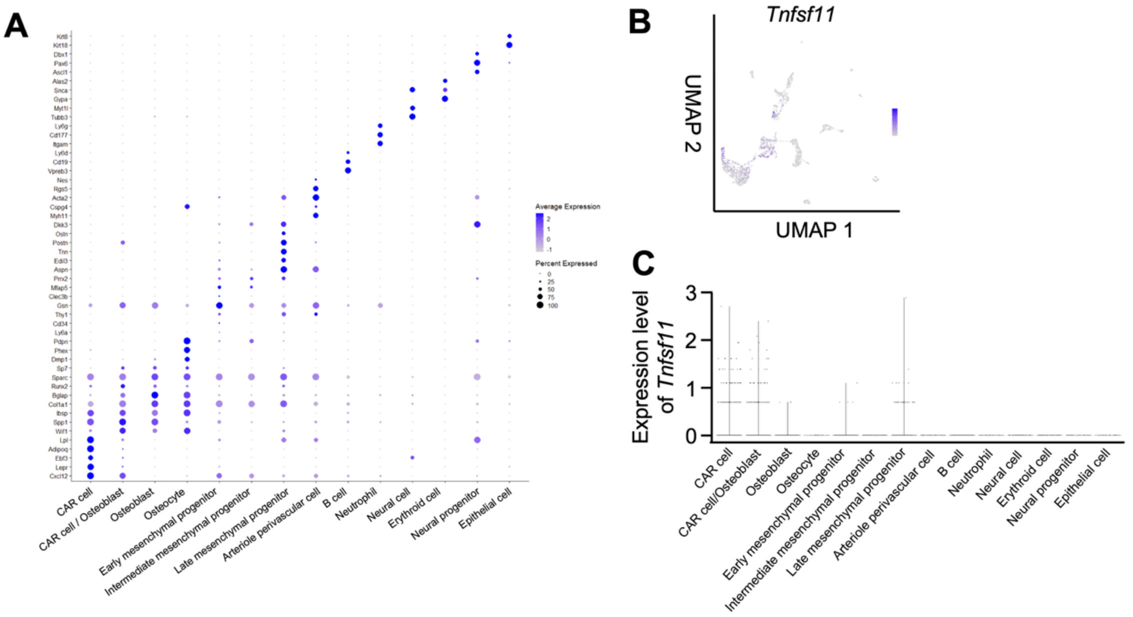

Fig. S11. The scRNA-seq analysis of mesenchymal cells.

(**A**) Dot pots showing the marker genes in each cell types. (**B**) Feature plots showing expression of *Tnfsf11*. (**C**) Violin plots showing the expression of *Tnfsf11* in each cell types.

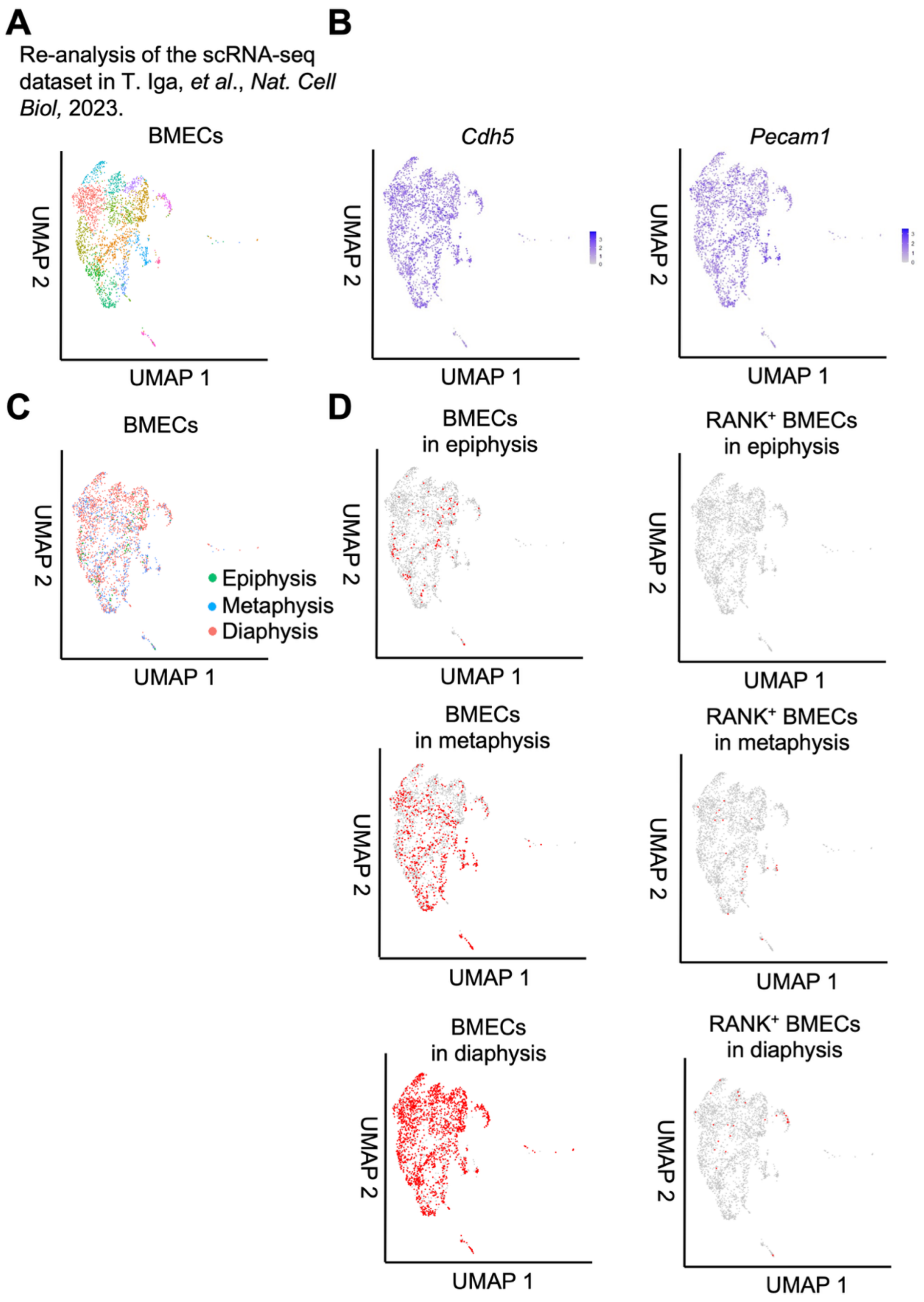

Fig. S12. Single-cell reanalysis of localized RANK expression in bone marrow endothelial cells.

We re-analyzed scRNA-seq datasets from T. Iga, et al. (4), in which bone marrow endothelial cells (BMECs) from the diaphysis, metaphysis, and epiphysis were individually examined.

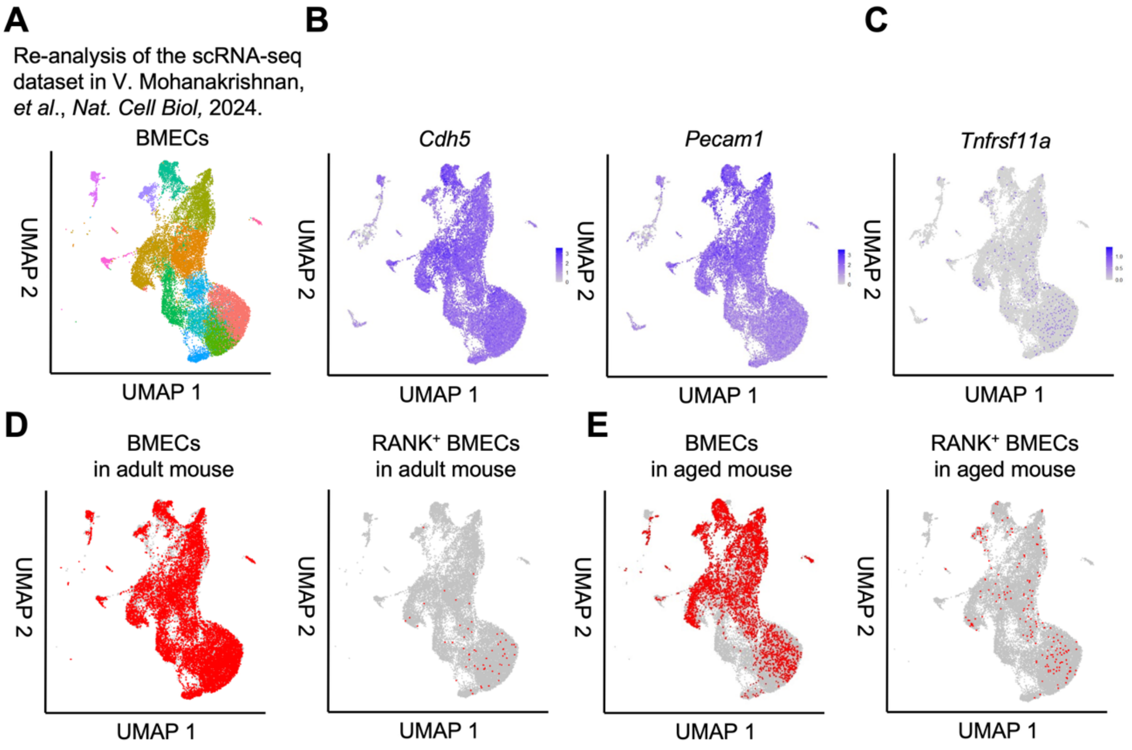

Fig. S13. Single-cell reanalysis of RANK expression in bone marrow endothelial cells during aging.

We reanalyzed single-cell RNA sequencing data from V. Mohanakrishnan, et al. (5) to investigate the effect of endothelial RANK expression during aging in the bone marrow.

Movie S1. Intravital imaging of high vascular leakage in the bone marrow.
Representative intravital multiphoton images of bone tissues from wild-type mice injected with fluorescein isothiocyanate-conjugated dextran [70 kDa (left panel) and 2,000 kDa (right panel)]. Blood vessels are shown in green, and bones are shown in blue. Scale bar: 50 μm. Playback speed: 150×.

Movie S2. Intravital imaging of the continuous entrance of monocytoid cells into the bone marrow.
Representative intravital multiphoton images of bone tissues from control (left panel) and RANKL-induced osteoporotic (right panel) LysM-EGFP mice injected with Alexa Fluor 594-conjugated isolectin IB4. Blood vessel walls are shown in red, and EGFP-positive cells are shown in green. Scale bar: 10 μm. Playback speed: 30×.
